# Study on signal transmission mechanism of arbuscular mycorrhizal hyphal network against root rot of *Salvia miltiorrhiza*

**DOI:** 10.1038/s41598-023-43278-5

**Published:** 2023-10-07

**Authors:** Song Han, Li Na, Zhang Rongchao, Hu Xiuqin, Zhang Wenyu, Zhang Bo, Li Xinpeng, Wang Zhen, Xin Jie

**Affiliations:** 1 School of Medicine, Linyi University, Linyi, 276000 Shandong China; 2 School of Chemistry and Chemical Engineering, Linyi University, Linyi, 276000 Shandong China; 3 Shandong New Era Pharmaceutical Co., Ltd., Fei County, 273400 Shandong China

**Keywords:** Plant biotechnology, Plant cell biology, Plant stress responses

## Abstract

To explore the signal transmission mechanism of the arbuscular mycorrhizal network against root rot of *Salvia miltiorrhiza*. In this experiment, the arbuscular mycorrhizal hyphal network was established among *Salvia miltiorrhiza* plants, and a two plant three-compartment culture model was established. The root of the donor *Salvia miltiorrhiza* was inoculated with the pathogenic fungi *Fusarium solani*. The changes of hormone signals such as jasmonic acid and salicylic acid and the expression of related defense genes in the recipient *Salvia miltiorrhiza* plants in different periods were measured, to study the underground disease resistance signal transmission mechanism among medicinal plants. *Salvia miltiorrhiza* can transmit the signal of resistance to root rot through the jasmonic acid pathway; When plants suffer from disease stress, the content of JA increases significantly, and the increase of JA content will inhibit the content of SA in plants; The gene expression of *PR-10* gene in the roots of *Salvia miltiorrhiza* with arbuscular mycorrhizal network infected by pathogenic fungi was 17.56 times higher than that inoculated only with pathogenic fungi; Changes in hormone content will also cause changes in the expression of related defense genes, such as *SnRK2* is inhibited by ABA in the signal transduction pathway, while JA and ABA show antagonistic changes after inoculation of pathogenic fungi in *Salvia miltiorrhiza*, so JA may positively regulate the expression of *SnRK2* gene. Plants can transmit signals through AM hyphal network after being stressed by the pathogen *Fusarium solani*. In the arbuscular mycorrhizal hyphal network, JA has important significance for the signal transmission of resistance to root rot and disease resistance of *Salvia miltiorrhiza*, which can make *Salvia miltiorrhiza* ready for stress resistance and improve the stress resistance of *Salvia miltiorrhiza*. *T*his experiment is of great significance to further analyze the signal transmission mechanism of the arbuscular mycorrhizal hyphal network.

## Introduction

*Salvia miltiorrhiza* bge. (Can be abbreviated as *S. milliorrhiza*) is a widely used traditional Chinese medicine in China. Its rhizome is used as medicine and has medicinal values such as activating blood circulation and removing blood stasis^1^, improving heart function^2,3^, and delaying aging^4^. With the research on the pharmacological effects of *S. miltiorrhiza*, the market demand for *S. miltiorrhiza* is increasing, the planting regulations of *S. miltiorrhiza* are expanding, and the phenomenon of root diseases and pests caused by continuous cropping is becoming more prominent. Relevant studies showed that with the gradual rise of the prevalence of root rot of *S. miltiorrhiza*, the yield, and quality of *S. miltiorrhiza* showed a significant downward trend^5,6^. Therefore, the soil-borne diseases mainly caused by the root rot of *S. miltiorrhiza* have seriously endangered and restricted the further development and utilization of *S. miltiorrhiza* resources.

Arbuscular mycorrhiza (AM) fungi are a kind of soil fungi that widely exist in nature and coexist with plants^7^. Studies have shown that inoculation of AM fungi on plants significantly improves the disease resistance of host plants^8^, and has a certain inhibitory effect after the occurrence of soil-borne diseases on plants^9^. Plants can combine with AM fungi to form mutualistic symbionts^10^. The extraradicular hyphae of AM fungi can infect the adjacent roots of *S. miltiorrhiza* and carry out hyphal fusion. The adjacent roots of *S. miltiorrhiza* are connected by extraradicular hyphae, thus forming arbuscular mycorrhizal networks (AMNS)^11^.

Plants will face various survival problems in the growth and development stage, such as abiotic stresses, cold, and drought and biological stresses such as microorganisms, diseases, and pests, which affect the growth and reproduction of plants. Plants have evolved a series of signal transmission and defense mechanisms in response to different stresses. Studies have shown that salicylic acid (SA) and jasmonic acid (JA) play important regulatory roles in plant biological stress responses. For example, when plants are fed by insects, a large amount of jasmonic acid (JA) will rapidly accumulate in the plant body, forming a conjugate JA ILE with isoleucine. JA IIE active molecules act on the JA pathway, promoting the receptor to combine with proteins to form a complex, and promoting the expression of downstream insect resistance-related genes to change in response to insect feeding and reduce damage to plants^12^. SA usually participates in defense responses against pathogenic fungi, including PTI (PAMP-triggered immunity) pathway and ETI (effector triggered immunity) pathway^13,14^. At the same time, SA is an essential signal molecule for SAR (systemic acquired resistance). Plants can respond to SA signals and rapidly initiate immune defense after being stimulated by pathogenic fungi^15,16^. Abscisic acid (ABA) can affect the transcriptional and post transcriptional modification of downstream regulators in the ABA signaling pathway, control plant responses to biological stress, and enhance plant resistance^17,18^. This shows that hormones play an important role in plant signal transmission, but the signal transmission mechanism of arbuscular mycorrhizal fungi under soil-borne disease stress is less studied at present.

When plants are under stress, pathogenesis-related proteins (PR) in plants will be produced and accumulated. PR proteins form an important part of the plant defense system. Plant hormones and disease resistance signals have a certain effect on the expression of *PR* genes. In different plants, *PR* genes are induced by different hormones. For example, studies have shown that when exogenous hormone MeJA is applied to bamboo root ginger, the activity of PR protein in bamboo root ginger seedlings inoculated with pathogenic fungi Ralstonia solanacearum is rapidly enhanced, which improves the disease resistance of bamboo root ginger^19^; when cassava was infected by whiteflies, various PR genes in the body showed different changes in response to whiteflies infection, and these *PR* genes were largely regulated by SA and JA^20^. After the infection of potato late blight and melon downy mildew on potato and melon, exogenous application of JA and SA can induce potato and melon to produce PR protein and other proteins, which is helpful for plants to improve disease resistance^21^. SnRK2 is only a protein kinase in plants. When ABA binds to the receptor protein to form a complex, SnRK2 protein kinase is activated and participates in the process of plant stress resistance^22^. Therefore, the study of the *SnRK2* gene is of great significance for the study of ABA signal transduction in mycorrhizal symbiotic plants. In this experiment, plant endogenous hormones JA, SA, and ABA were used as detection hormones. The expression levels of the disease-related gene *PR-10* and the *SnRK2* gene in the differentially expressed genes annotated in the transcriptome data were studied under different conditions of inoculation with AM fungi and pathogenic fungi.

In this experiment, the AM hyphal network was established among *S. miltiorrhiza* plants. By setting up a two plant three compartment culture model, the root of donor *S. miltiorrhiza* was inoculated with the pathogenic fungi *Fusarium solani*, and the changes of hormone signals such as JA and SA and the expression of related defense genes in recipient *S. miltiorrhiza* plants in different periods were measured, to study the underground disease resistance signal transmission mechanism among medicinal plants, It provides a theoretical basis for the use of beneficial microorganisms mycorrhizal fungi for biological control of diseases and the breeding of genuine Chinese medicinal materials.

## Material and methods

### Test material and reagents

The test plant material is *Salvia miltiorrhiza* Bunge, purchased from the *S. miltiorrhiza* planting base in Junan County, Shandong Province. The tested AM fungus is Streptomyces mosaicus, purchased from the Root Biology Research Institute of Changjiang University. The test pathogen was *Fusarium solani*, which was ordered from China Center for Type Culture Collection (CCTCC AF 2014003).

The plant culture medium is composed of organic nutrient soil (pH = 5.5–7.0, organic matter content ≥ 40%) and ordinary soil on the campus of Linyi University, mixed in a ratio of 2:1 and then packaged. The soil is sterilized by high-pressure steam at 121 °C for 1 h to eliminate fungal spores. After sterilization, it is cooled down for later use.

JA standard and ABA standard were purchased from Sigma; SA standard was purchased from Dr.ehrenstorfer company; Deuterated salicylic acid (D-SA) standard and deuterated abscisic acid (D-ABA) standard were purchased from Olchemim; Dihydrojasmonic acid (2HJA) standard was purchased from TCI company; PDA medium and PD medium were purchased from Haibo biological company; Hydrochloric acid, isopropanol, dichloromethane, methanol and formic acid are domestic or imported chromatographic pure (GR) reagents; DNA marker was purchased from Takara company; Supergreen/gelgreen nucleic acid dye was purchased from biosharp; 6 × DNA loading buffer was purchased from Solarbio; Agarose was purchased from yeasen company; Trilyl benzene blue stain was purchased from volkey biological Co., Ltd. Potassium hydroxide and ethylenediamine tetraacetic acid are analytical pure (AR) reagents purchased from Hengxing chemical reagent company; Anhydrous ethanol, hydrochloric acid, glycerol, lactic acid, chloroform, isopropanol and glacial acetic acid are analytical pure (AR) reagents purchased from Sinopharm Chemical Reagent Co., Ltd.

### Experimental design

In the experiment, a two plant three chamber culture model was used. The bottom of a container was divided into three-chambers. The donor *S. miltiorrhiza* and the recipient *S. miltiorrhiza* were planted, and the donor *S. miltiorrhiza* was cultured in a single plant two-chamber culture. Four treatment groups A, B, C, and D, were established based on whether the *S. miltiorrhiza* plants were symbiotic with AM fungi, whether the AM hyphal network was established between the donor and the recipient, and whether the donor *S. miltiorrhiza* was applied with the pathogen *Fusarium solani.*

- Group A: the donor and recipient of S. miltiorrhiza were inoculated with AM fungi, and the hyphal network was connected to impose pathogenic fungi on the donor S. miltiorrhiza;
- Group B: the donor and recipient of *S. miltiorrhiza* were completely isolated without a hyphal network connection, and pathogenic fungi were applied to the donor *S. miltiorrhiza*;
- Group C: the donor and recipient of *S. miltiorrhiza* were inoculated with AM fungi, with hyphal network connection, but no pathogenic fungi were applied to the donor *S. miltiorrhiza*;
- Group D: the donor and recipient of *S. miltiorrhiza* were not inoculated with AM fungi, there was no hyphal network between them, and pathogenic fungi were applied to the donor *S. miltiorrhiza*.

In this experimental model, the left side is the donor (G) *S. miltiorrhiza*, and the right side is the recipient (ST) *S. miltiorrhiza*. The root system of the donor *S. miltiorrhiza* is divided into two chambers, written as G1 and G2 respectively, and the recipient *S. miltiorrhiza* is written as ST. To exclude the influence of leaf volatiles, the aboveground part of *S. miltiorrhiza* was covered and sealed by self-sealing bags during the experiment.

### AM hyphal network construction

**Figure 1:**
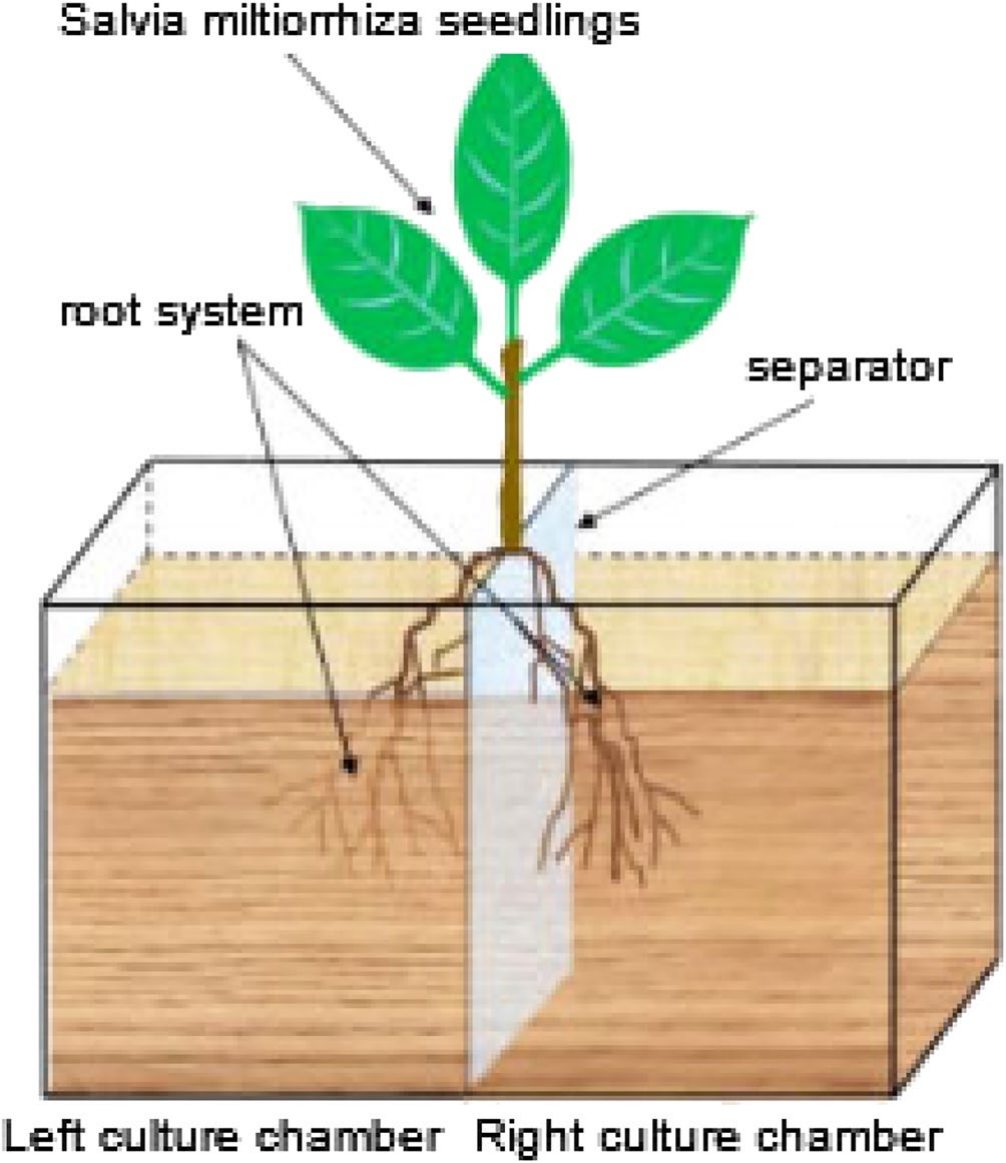
Single-compartment culture model.

In the test, the donor *S. miltiorrhiza* was cultured in a single plant with two chambers. The root system of a donor *S. miltiorrhiza* was divided into two parts, which were placed in two culture chambers, separated by plastic partitions. See Fig. 1. Both sides of the culture chamber were inoculated with AM fungi, 45 d later, the pathogen *Fusarium solani* was applied to the left root of the donor *S. miltiorrhiza*, and the changes of hormone content in the root system of *S. miltiorrhiza* on both sides over time were detected.

**Figure 2:**
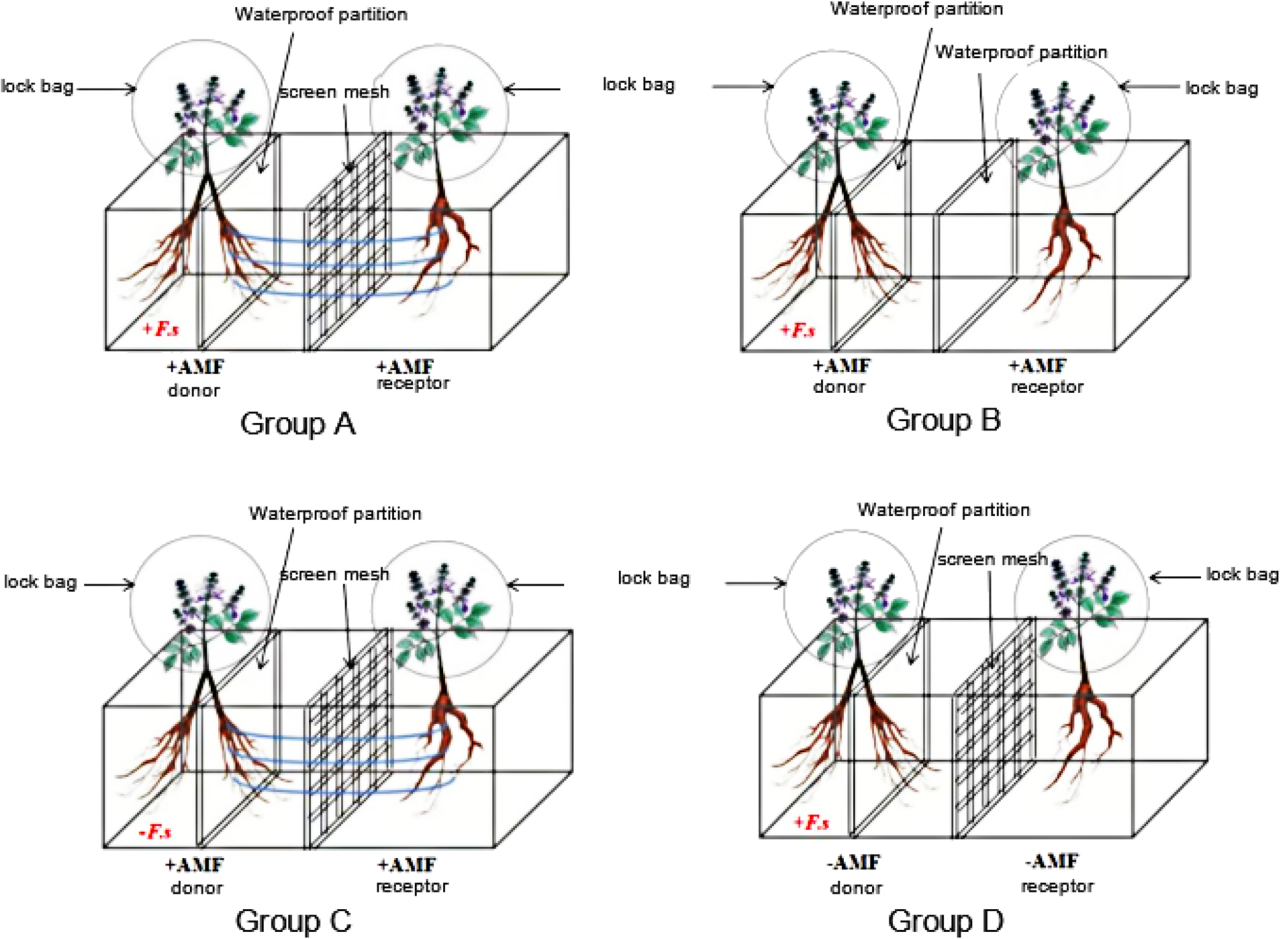
Model for signaling studies of *Salvia miltiorrhiza*. Figure shows a signal transduction research model of *S. miltiorrhiza*, divided into four groups. Each basin of *S. miltiorrhiza* consists of two *S. miltiorrhiza* plants. The left *S. miltiorrhiza* is the donor *S. miltiorrhiza*, and the root is divided into two parts using a waterproof partition. The left part is marked as G1, and the right part is marked as G2; The other *S. miltiorrhiza* is the recipient *S. miltiorrhiza*, separated from the donor *S. miltiorrhiza* by a sieve or waterproof partition, denoted as ST. AMF was applied to the soil of groups A, B and C flowerpots, while group D *S. miltiorrhiza* did not apply AMF. At the same time, *Fusarium solani* FS was applied to A-G1, B-G1, and D-G1. Seal the above ground part with a self sealing bag.

To test whether the arbuscular mycorrhizal hyphal network can mediate the signal transmission of resistance to root rot among *S. miltiorrhiza* plants, four treatment groups were set up, as shown in Fig. 2. In group A, 35 μM screen separation for AM fungal hyphae to pass through; In group B, the two strains of *S. miltiorrhiza* were completely separated by a waterproof partition. The donor and recipient of *S. miltiorrhiza* were in an independent space. Although both of them were symbiotic with AM fungi, there was no hyphal network connection; In group C, the donor and recipient of *S. miltiorrhiza* were separated by a mesh, and a hyphal network could be formed between the two plants; In group D, the two strains of *S. miltiorrhiza* were separated by a screen and were not inoculated with AM fungi, so there was no hyphal network connection.

### Planting, management and experimental treatment

The bottom of the planting box was drilled and washed with running water, dried, and disinfected with 75% alcohol. Pour the sterilized and cool dry mixed culture medium into the incubator, and plant healthy *S. miltiorrhiza* seedlings with uniform size. Three replicates in each group were randomly placed. After planting for 1 d, water it thoroughly. After the soil is dry, water 1 L every 5 d. Use concentrated seaweed nutrient solution at the ratio of 1:200, and supplement it every 15 d. After 4 months of growth of *S. miltiorrhiza*, 50 g of AM fungi were inoculated into the three chambers of *S. miltiorrhiza*, and the same amount of sterilized fungi was inoculated into the control. After 40 d of inoculation, the infection rate of AM fungal roots was detected by the trypan blue staining method. On 45 d, the root of *S. miltiorrhiza* was inoculated with 20 mL of Fusarium Pythium spore suspension (1.0 × 10^7^ CFU), and the control was replaced with an equal amount of dd H_2_O^23^.

The sampling time of materials was set as five time periods. The pathogenic fungi *Fusarium solani* was applied to *S. miltiorrhiza* and set at 0 h. The root system of *S. miltiorrhiza* was sampled at 24 h, 72 h, 120 h, 144 h, and 192 h^24^.

### Detection of AM fungal infection rate

The root system of *S. miltiorrhiza* Bunge was stained by using the method of staining with triphenyl blue. The root system was added to the 5% KOH solution. After the 90 °C water bath, the waste liquid was discarded to clean the root system, 2% HCl solution was added, and the root system was placed at room temperature for 5 min. the waste liquid was discarded to clean the root system, 0.05% triphenyl blue was added, and the 90 °C water bath was 30 min; Discarded the waste liquid to clean the root system, added the decolorizing solution, decolorized at room temperature, and stood overnight^25^. Finally, the mycorrhizal infection rate was calculated using the conventional method of root segment frequency.

### Hormone content determination

Preparation of test article: take out the sample stored at -80 °C and grind it to dry powder in liquid nitrogen; Add 7 mL of isopropanol water hydrochloric acid mixed extraction solution to the glass test tube (2-propanol/H20/concentrated HCl(2:1:0.002,vol/vol/vol)); Add 8 µL of 1 µg/mL internal standard solution and shake at 4 °C for 30 min; Add 8 mL of dichloromethane, shake at 4 °C for 30 min, centrifuge at 13,000 r/min for 5 min, and take the lower organic phase;

Protected from light, the organic phase was blown dry with nitrogen and redissolved with methanol (0.1% formic acid); Centrifuge at 4 °C for 10 min (13,000 g), take the supernatant and filter through a 0.22 µM filter membrane, and detect it by HPLC–MS/MS.

Standard solution preparation. The standard solutions of SA, JA, and ABA with gradients of 0.1 ng/mL, 0.2 ng/mL, 0.5 ng/mL, 2 ng/mL, 5 ng/mL, 20 ng/mL, 50 ng/mL, and 200 ng/mL were prepared with methanol (0.1% formic acid) as solvent, and the internal standard solution with final concentration of 20 ng/mL was added.

The data acquisition system mainly includes high-performance liquid chromatography (HPLC) (Agilent 1290) and tandem mass spectrometry (MS/MS) (Applied Biosystems 6500 quadrupole trap). Mass spectrometry data were processed using the software analyst, the specific parameters are shown in Table 1.

**Table 1:** Selected reaction monitoring conditions for protonated or deprotonated plant hormones ([M + H]^+^ or [M − H]^−^).

Substance name	Parent ion (m/z)	Quantitative ion (m/z)	Qualitative ion (m/z)	De clustering voltage (V)	Collision energy (V)
SA	137.0	92.9	65.0	− 50	− 20/− 39
JA	209.2	58.9	58.9	− 54	− 16
ABA	263.1	153.0	204.2	− 60	− 14/− 27

Drawn a standard curve for hormones used in the internal standard method experiment and carried out methodological validation. The hormone standard curve and methodological validation data are shown in Table 2.

**Table 2:** Hormone name, Linear range, Calibration curve, Correlation coefficient and weight of the phytohormones.

Class	Linear range (ng/mL)	Calibration curve	Correlation coefficient (r)	Weight
JA	0.1–200	y = 0.54797 x + 0.00121	(r = 0.99154)	1/x^2^
SA	0.2–200	y = 1.96046 x + 0.07376	(r = 0.99492)	1/x^2^
ABA	0.1–200	y = 25.80258 x + 0.07812	(r = 0.99514)	1/x^2^

### Relative gene expression assay

Use the Sangon Biotech Spin Column Plant Total RNA Purification Kit (NO.: B518661) to extract total RNA from the root system, Store at -80 °C. Perform integrity, quality, and concentration tests on RNA, and synthesize cDNA using the Dongyang Spin Reverse Transcription Reaction Kit for RNA that meets quality requirements. Based on transcriptome data from previous experiments, specific primers were designed for the *PR-10* gene and *SnRK2* gene sequences using Primer Premier 5 software, and the *Actin* gene was selected as the internal reference gene^26^. The primer sequences are shown in Table 3. The RT-PCR reaction system is 20 μL. Amplification program settings: 95 °C for 5 min, 95 °C for 10 s, 55 °C for 20 s, 72 °C for 20 s, 40 cycles, instrument default for melting curve stage.

**Table 3:** Primers used for genes in real-time PCR.

Gene	Primer sequences
*PR-10*	*PR-10*-F 5′ CCATTTCTCCAAATTCCAAGAG 3'
	*PR-10*-R 5′ CCTCGTCTCTTTCAGTAGTC 3'
*SnRK2*	*SnRK2*-F 5′ GTCACTCAGACACCCCAAT 3'
	*SnRK2*-R 5′ TTCACTAAATCTCCCAGCA 3'
*Actin*	*Actin*-F 5′ AGGAACCACCGATCCAGACA 3'
	*Actin*-R 5′ GGTGCCCTGAGGTCCTGTT 3'

### Data statistics

In this study, we used a T-test to compare the differences in hormone levels between different sampling times. Data was recorded and calculated using Microsoft Excel 2013 (Office 15), followed by Standard deviation analysis using Excel, GraphPad Prism 9 software and mean comparison method, significance analysis plotting using GraphPad Prism 9 software.

### Ethical approval

We confirm that all methods are carried out in accordance with the relevant guidelines in the methods section. Meanwhile, the use of medicinal plants in this experiment complies with relevant institutions, national and international guidelines and legislation.

## Result and analysis

### Infection rate of AM fungi in the root system of *S. miltiorrhiza*

#### Detection of Trimethylbenzene blue staining

**Figure 3:**
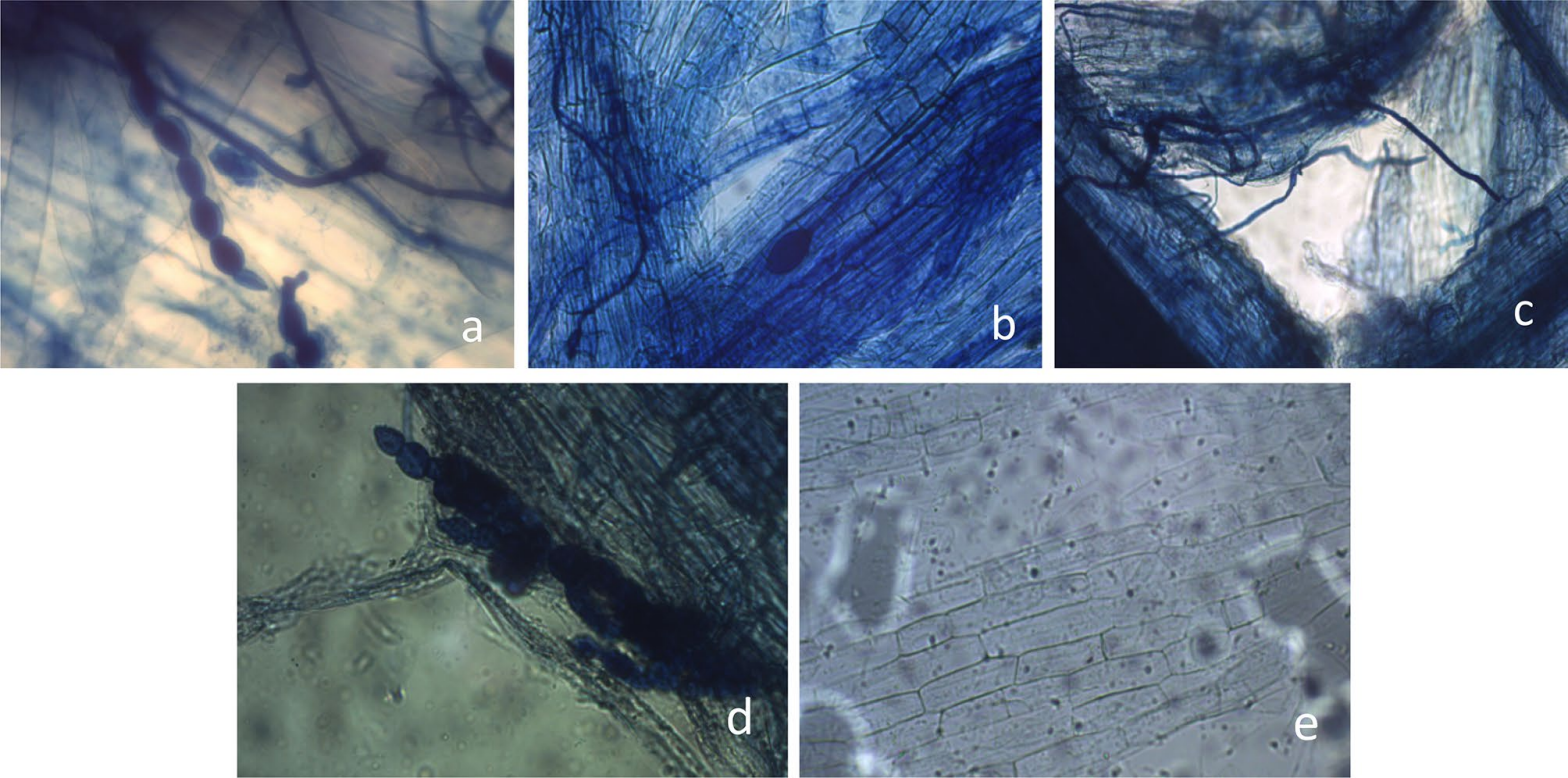
*Salvia miltiorrhiza* is involved in the symbiotic mycorrhizal staining map in AM fungi. Note: (**a**–**d**) show the root system of *S. miltiorrhiza* with AMF applied; (**e**) shows the root system of *S. miltiorrhiza* without AMF applied (10 × ,10 ×).

After sampling the root system, a staining experiment was conducted using triphenyl blue to observe whether AM fungi had successfully infected the root system of *S. miltiorrhiza*. Treatment groups A, B, and C were treated with AM fungi, while group D was not inoculated with AM fungi. Observe the root systems of the four groups under a microscope by staining them with a triphenyl blue reagent. As shown in Fig. 3, spores and endophytic hyphae can be seen in Fig. 3a and b, while Fig. 3c contains extracellular hyphae, Figure d contains spores, while Figure e does not have these structures.

#### Calculate the AM fungal infection rate of the *S. miltiorrhiza* root system

As shown in Table 4, after 40 d of AM fungi inoculation, the root system of four groups of *S. miltiorrhiza* donors and recipients were sampled, and the infection rate of AM fungi was calculated by observing whether the AM fungi successfully infected the root system of *S. miltiorrhiza* through the triphenyl blue staining experiment.

**Table 4:** Root AM fungal infection rates of *Salvia miltiorrhiza.*

Group	G1 root AM fungal infection rate (%)	G2 root AM fungal infection rate (%)	ST root AM fungal infection rate (%)
A	48.13	47.27	50.03
B	51.02	48.92	50.372
C	47.96	47.29	48.493
D	–	–	–

### Quality testing of total RNA of *S. miltiorrhiza*

Selecting RNA with clear and bright electrophoretic bands, good quality, and high integrity for later experiments.

### Changes in hormone content in the root system of *S. miltiorrhiza* after AMF treatment alone

In the research model of signal transduction in *S. miltiorrhiza*, after applying *Fusarium solani* to B-G1, *Fusarium solani* can be transmitted to B-G2 through the roots. However, due to the presence of waterproof barriers between B-ST and B-G2, will hinder the transmission of *Fusarium solani* between B-ST and B-G2. Therefore, a mycelial network cannot be formed between B-ST and B-G2. In group D, although *Fusarium solani* can be transmitted from D-G1 to D-G2 through the root of *S. miltiorrhiza*, and the screen between D-G2 and D-ST can carry out signal transmission, due to the absence of AMF fungi in the soil, a mycelium network cannot be formed between D-G and D-ST.

**Figure 4:**
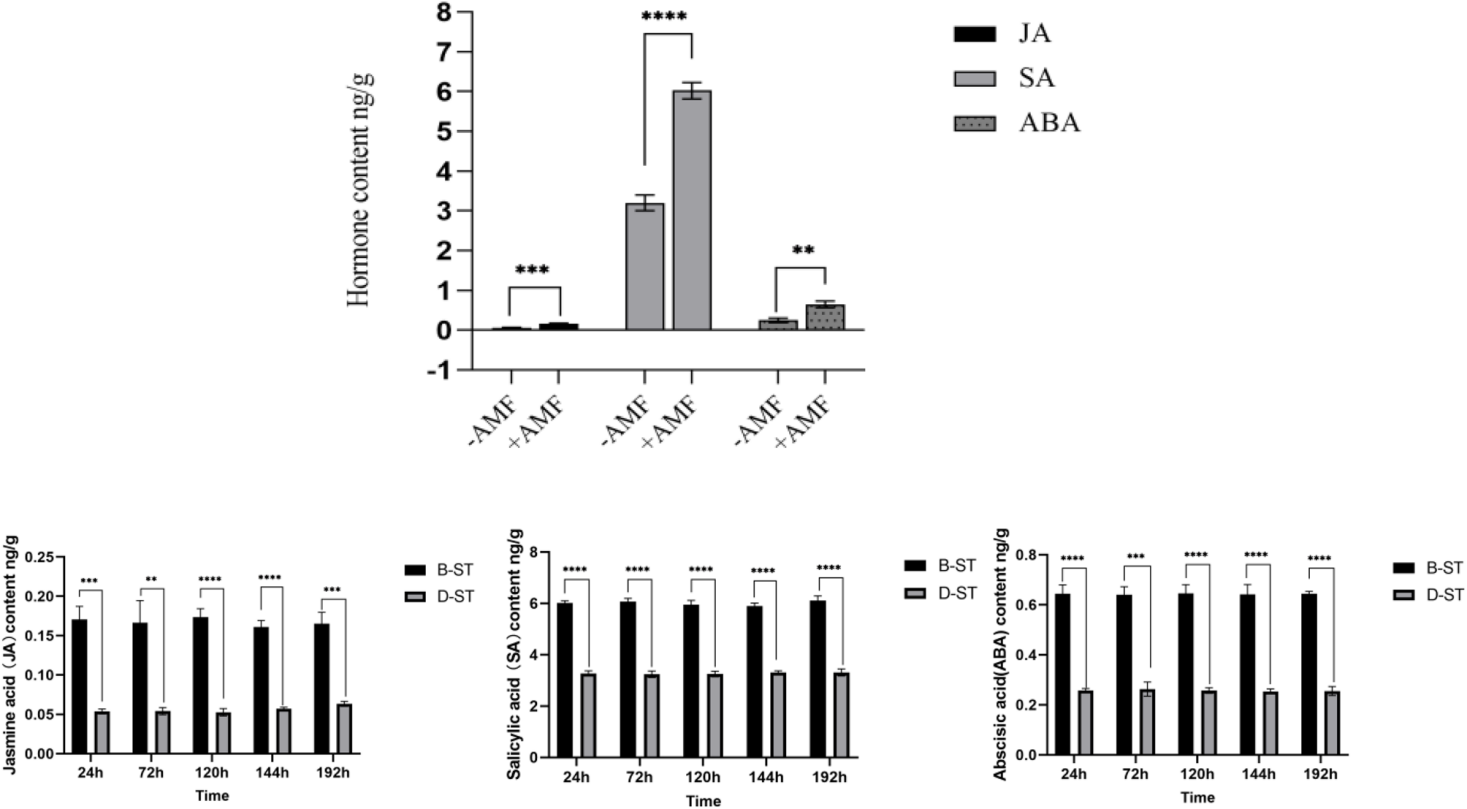
Changes in hormone content in *Salvia miltiorrhiza* with AMF versus without AMF. ****p ≤ 0.0001, ***p ≤ 0.001.

As can be seen in Fig. 4, B-ST is symbiotic due to the application of AM fungi. Without receiving external stimuli, the contents of JA, SA, and abscisic acid (ABA) of *S. miltiorrhiza* itself were significantly higher than those of D-ST without AM fungi. The JA, SA, and ABA contents of B-ST are 2.98 times, 1.84 times, and 2.50 times of D-ST respectively.

Under the stimulation of no pathogenic fungi, the hormone content of *S. miltiorrhiza* with AM fungi was much higher than that of *S. miltiorrhiza* without AM fungi, which indicated that a certain amount of endogenous hormones would accumulate after the participation of *S. miltiorrhiza* in the symbiosis of AM fungi. JA^27^, SA^28^, and ABA^29^ as important signaling molecules in the process of plant disease resistance, are closely related to various physiological and biochemical reactions of plants. The accumulation of JA and SA can effectively improve the disease resistance of plants^30^. The accumulation of ABA can also improve the ability of plants to resist stress. ABA signaling also plays an important role in plant disease resistance. Therefore, the symbiosis of AM fungi and *S. miltiorrhiza* can effectively improve the resistance of *S. miltiorrhiza* and the tolerance to diseases. C-GT and C-ST applied AM fungi, but were not inoculated with *Fusarium solani*, so the change of hormone content is consistent with that of B-ST, which can be used to exclude the influence of AM fungi and *S. miltiorrhiza* after forming mycorrhizas.

### Changes of hormone content in the roots of *S. miltiorrhiza* treated with AMF and *Fusarium solani*

#### Changes of JA content in root system of *S. miltiorrhiza*

**Figure 5:**
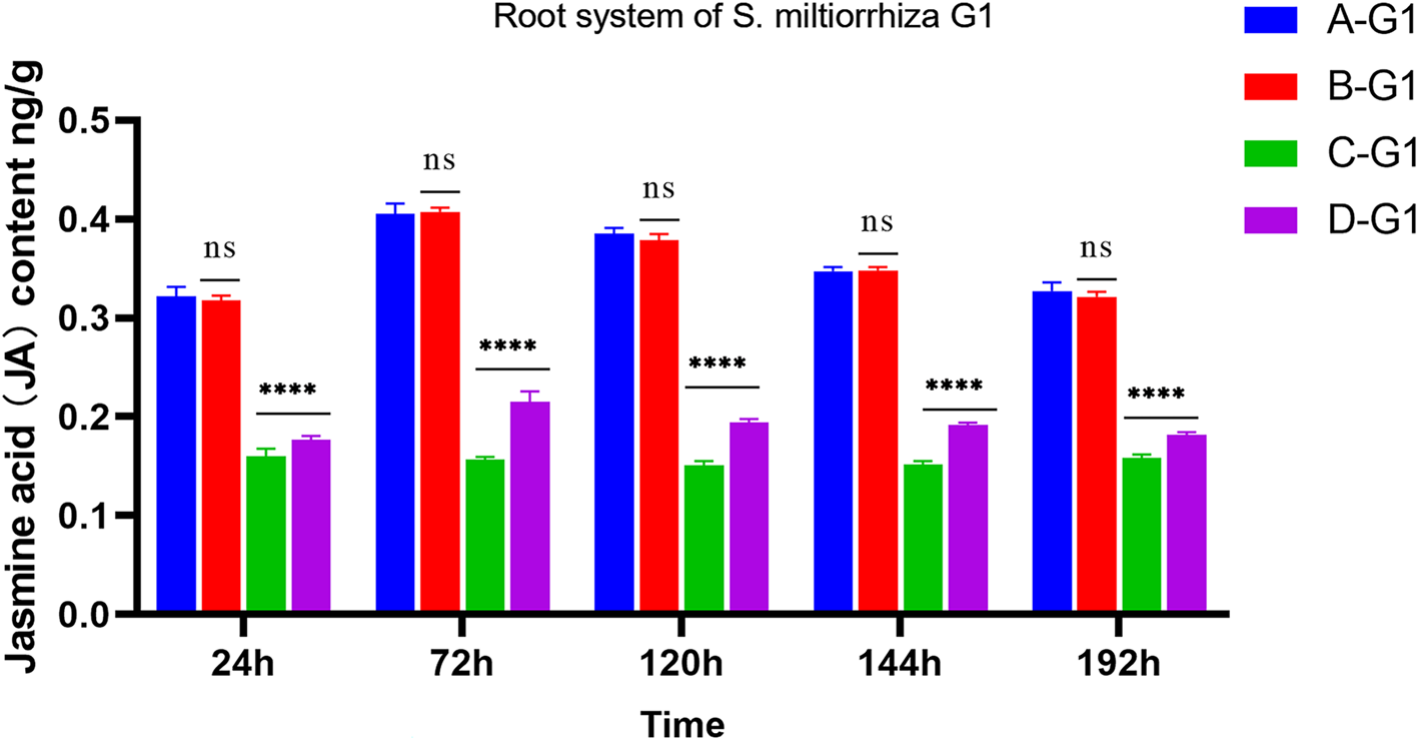
JA content in the root system of donor G1. ****p ≤ 0.0001, ^ns^*p* > 0.05.

The difference between A-G1, B-G1, and C-G1 is that A-G1 and B-G1 apply pathogenic fungi, while C-G1 does not. From the expression level of JA in Fig. 5, it can be seen that the JA content of C-G1 without the application of pathogenic fungi is stable, while the JA content of A-G1 and B-G1 shows a trend of first increasing and then decreasing, indicating that the application of pathogenic fungi can indeed increase the JA content. Compared with D-G1, A-G1, B-G1 both applied pathogenic fungi, with the difference being that D-G1 did not apply AMF fungi. From Fig. 5, it can be seen that the variation trend of JA content in A-G1, B-G1, and D-G1 is consistent. However, it is evident that at the same time, the JA content in A-G1 and B-G1 is approximately twice that of D-G1 without the application of AMF.

After AM fungi were applied to group A, a hyphal network could be formed between A-G2 and A-ST for signal transmission. The JA content in donor G1 increases from 24 to 72 h, and the JA content of *S. miltiorrhiza* roots after applying pathogenic fungi showed a trend of first increasing and then decreasing, and the JA content of A-G1 was always higher than that of D-G1 without AM fungi. In group A, the JA content of G1 increased significantly at 72 h and then decreased slowly. However, through the analysis of G2, it was found that the JA content continued to increase from 24 to 120 h, and then decreased at 144 h, in the group with *Fusarium solani*, the change of hormone content in G2 lagged behind that in G1. It can be found that hormones play an important role in signal transmission. Meanwhile, this also indicates that *S. miltiorrhiza* can rapidly induce its own JA content to increase after infection with pathogenic fungi. The JA content of D-G1 infected by pathogenic fungi was higher than that of B-ST of *S. miltiorrhiza* inoculated with AM fungi. This is because when *S. miltiorrhiza* is invaded by pathogenic fungi, JA content as an important signal molecule in the process of plant disease resistance will significantly increase, thus playing a role in disease resistance in the root system^31^. The JA content of *S. miltiorrhiza* has been at a high level after being inoculated with AM fungi in advance. When the plant is under disease stress, it can rapidly increase JA content to enhance the resistance to root rot.

**Figure 6:**
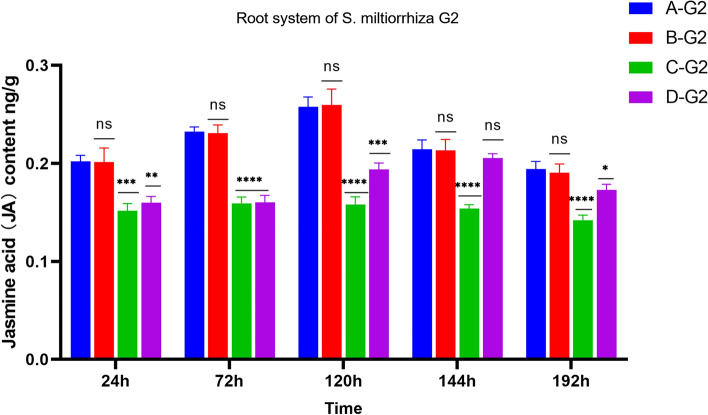
JA content in the root system of *Salvia miltiorrhiza* donor G2. ****p ≤ 0.0001, ***p ≤ 0.001, **p ≤ 0.01, *p ≤ 0.05, ^ns^*p* > 0.05.

As shown in Fig. 6, when A-G1 was subjected to disease stress, the JA content of the A-G2 *S. miltiorrhiza* root showed a trend of first increasing and then decreasing. At 120 h, the JA content of A-G2 reached the highest point, but at the same time, the JA content in A-G2 is always lower than that in A-G1. Compared with the JA content of D-G2 roots, the JA content of A-G2 roots was always higher than that of D-G2 without AM fungi inoculation, but the difference was not as obvious as that of A-G1, and the JA content of D-G2 reached the highest point at 144 h. This may be because the stress occurs in the G1 root system, but not directly in the G2 root system. When AM fungi are applied to *S. miltiorrhiza*, AM fungi and *S. miltiorrhiza* are symbiotic. Both B-ST and D-ST did not apply *Fusarium solani* fungi, but B-ST applied AMF fungi, while D-ST did not. From the comparison of the hormone levels in Fig. 4, it can be seen that the three hormone levels in B-ST with AMF fungi were higher than those in D-ST. Therefore, Under the condition of not applying pathogenic fungi, JA accumulates in a certain amount in *S. miltiorrhiza*. When *S. miltiorrhiza* is under disease stress, the disease resistance signal can be transmitted to neighboring plants through the hyphal network, so that the JA content of neighboring plants increases rapidly, so that plants can prepare for disease defense in advance and cope with stress.

**Figure 7:**
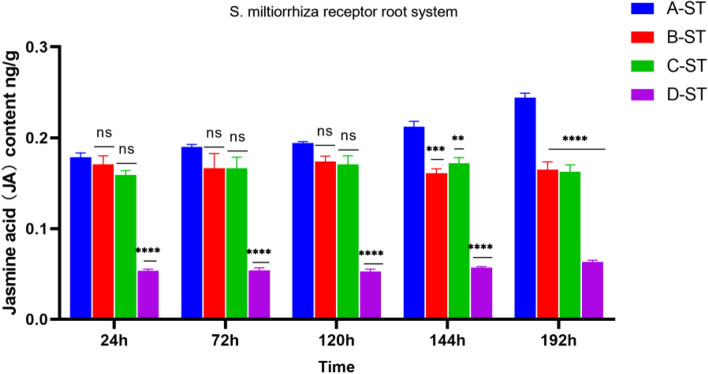
JA content in root system of *Salvia miltiorrhiza* receptor ST. Note: ****p ≤ 0.0001, ***p ≤ 0.001, **p ≤ 0.01, ^ns^*p* > 0.05.

**Figure 8:**
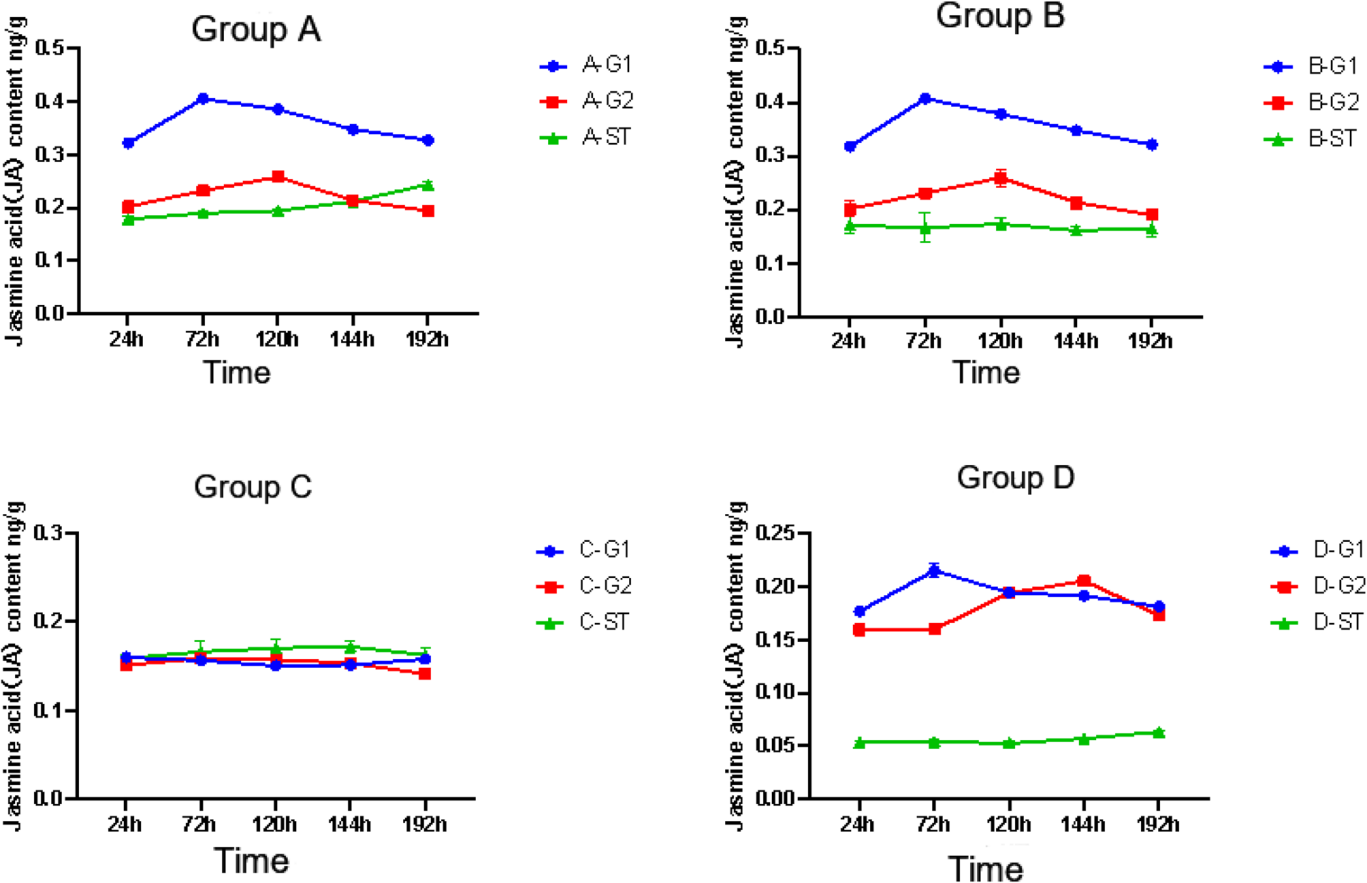
Changes in root JA content of *Salvia miltiorrhiza* in each group.

As shown in Figs. 7 and 8, after the pathogen infection treatment of *S. miltiorrhiza* donor G1, the receptor JA content in groups B, C, and D had been stably expressed without obvious change. Although A-ST was not directly threatened by the pathogen, it showed a trend of first stable change and then increasing., which indicated that *S. miltiorrhiza* was induced to activate JA in vivo after suffering from the pathogen *Fusarium solani*, and the JA content increased to resist stress. When there is a hyphal network connection between the donor and the recipient *S. miltiorrhiza*, JA can stimulate the recipient healthy *S. miltiorrhiza* to receive defense signals, so that JA in the recipient’s *S. miltiorrhiza* is activated and expressed, and the content gradually increases in response to stress. By comparing the JA content of B-ST with four samples of C-G1, C-G2, and C-ST, which were only treated with AMF fungi and not with *Fusarium solani* fungi, as shown in Fig. 8, the JA content remained unchanged, while B-G2 showed an increase from 24 to 120 h and a decrease thereafter. Moreover, the JA content of B-G1 reaches its peak at 72 h, while B-G2 only reaches its peak at 120 h, indicating that the transmission of disease resistance signals from B-G1 to B-G2 takes time. When one side of the root system is attacked by pathogenic fungi, the disease resistance signal will be transmitted from one side of the root system to the other side of the healthy root system, and then through the hyphal network, the disease resistance signal will be transmitted to the adjacent nonstressed plants. After receiving the disease resistance signal, the plants will make a defense mechanism in advance. In group D, no AM fungi were applied to *S. miltiorrhiza*, so there was no hyphal network connection between the recipient and the donor, and the JA content in D-ST showed a low-level change.

#### Changes in salicylic acid content in the root system of *S. miltiorrhiza*

**Figure 9:**
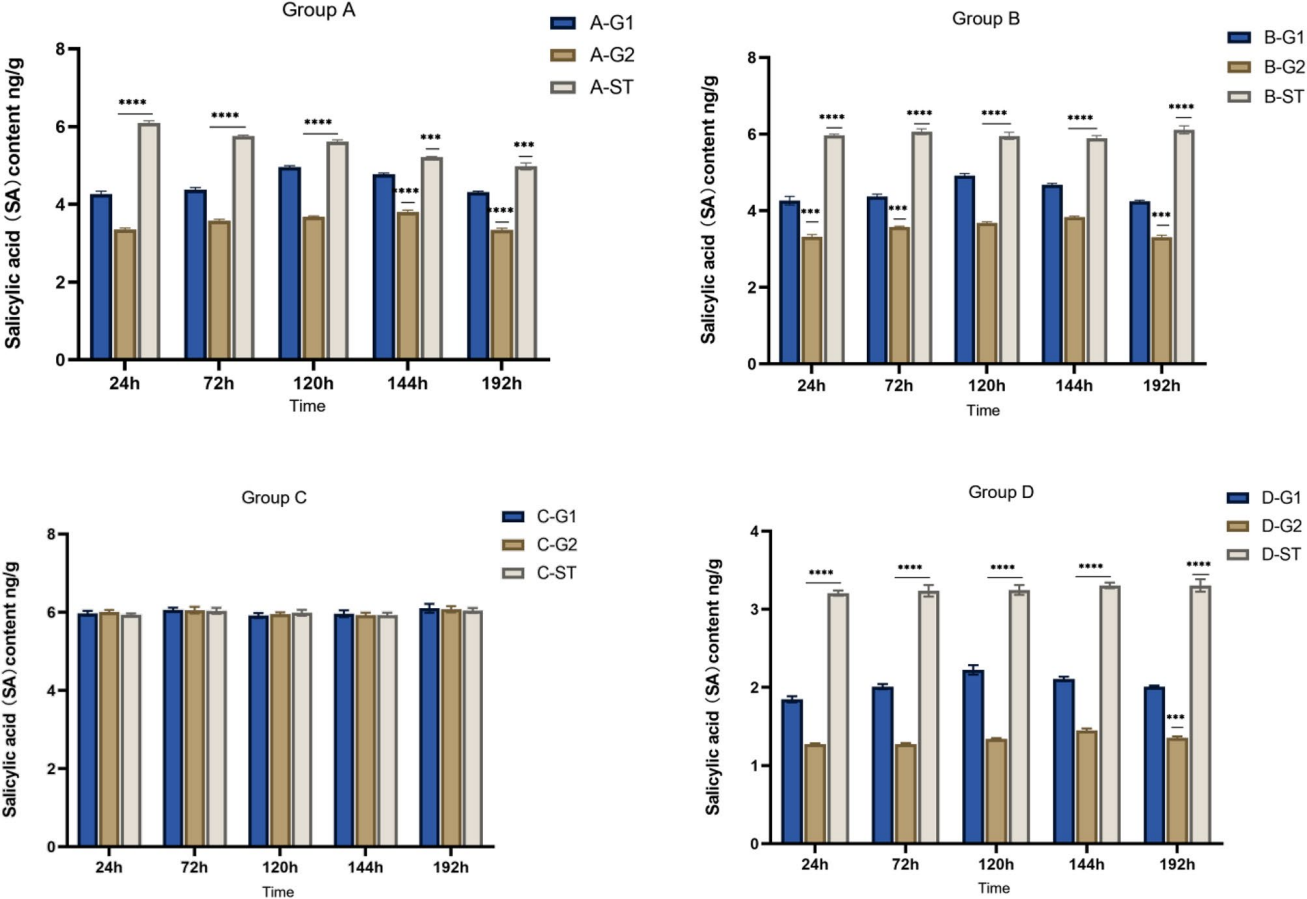
Changes of SA content in the root system of *Salvia miltiorrhiza* in each group. ****p ≤ 0.0001, ***p ≤ 0.001.

**Figure 10:**
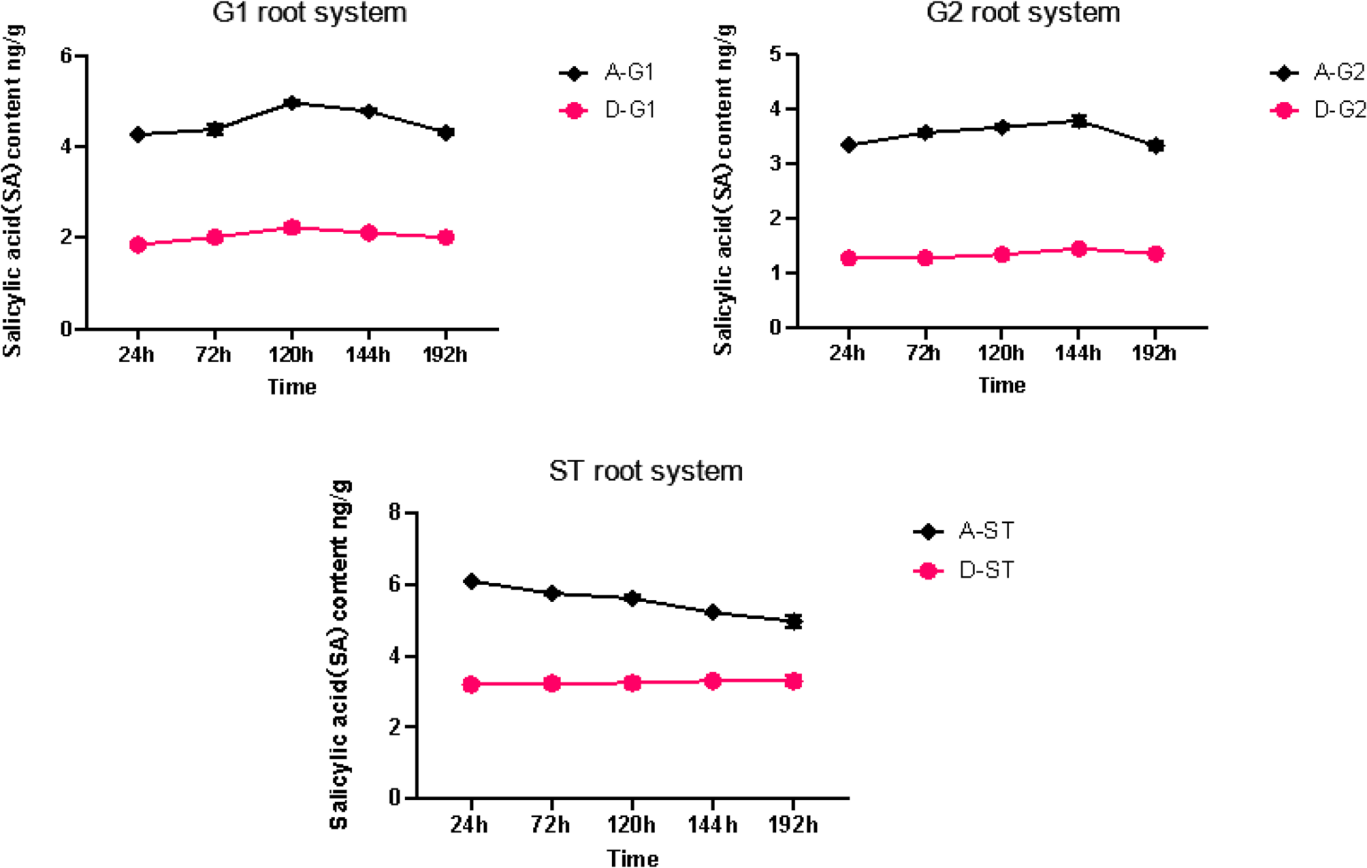
Comparison of root SA content between donor and recipient roots in A group and D group.

Group A was treated with AM fungi, while group D was not treated with AM fungi. The root system of *S. miltiorrhiza* of A-G1 and D-G1 were inoculated with *Fusarium solani*. The changing trend of SA in the roots of the donors exposed to pathogenic fungi stresses is similar to that of JA. As shown in Figs. 9 and 10, the change of SA content in the roots of both groups of *S. miltiorrhiza* donors in groups A and D shows a trend of first increasing and then decreasing, and the SA content in the roots of A-G1 is higher than that of A-G2, and that of D-G1 is higher than that of D-G2; SA content in roots of A-G1 and A-G2 with AM fungi was higher than that of D-G1 and D-G2 without AM fungi.

However, different from the changes in JA, the content of SA in *S. miltiorrhiza* after the application of pathogenic fungi was lower than that of *S. miltiorrhiza* without pathogenic fungi, which may be due to the antagonistic effect of JA and SA. When plants suffer from disease stress, the content of JA increases significantly, and the increase of JA content will inhibit the content of SA in plants^32^ . Therefore, after the application of pathogenic fungi, the content of SA in *S. miltiorrhiza* is lower than that without pathogenic fungi.

There is a hyphal network between the recipient and the donor of *S. miltiorrhiza* in group A, so A-ST can receive the disease resistance signal transmitted by the donor *S. miltiorrhiza*. Before the disease resistance signal is transmitted to the recipient, the SA content of A-ST is not much different from that of B-ST inoculated with AM fungi alone. Due to the increase of JA content of the recipient *S. miltiorrhiza*, the SA content in the recipient *S. miltiorrhiza* shows an upward trend, but due to the antagonistic effect of JA, the content is lower than that of *S. miltiorrhiza* inoculated with AM fungi alone.

### Induced expression of genes in roots of *S. miltiorrhiza* after CO-treatment of AMF and *Fusarium solani*

#### Induced expression of *PR-10* gene in the roots of donor and recipient *S. miltiorrhiza*

**Figure 11:**
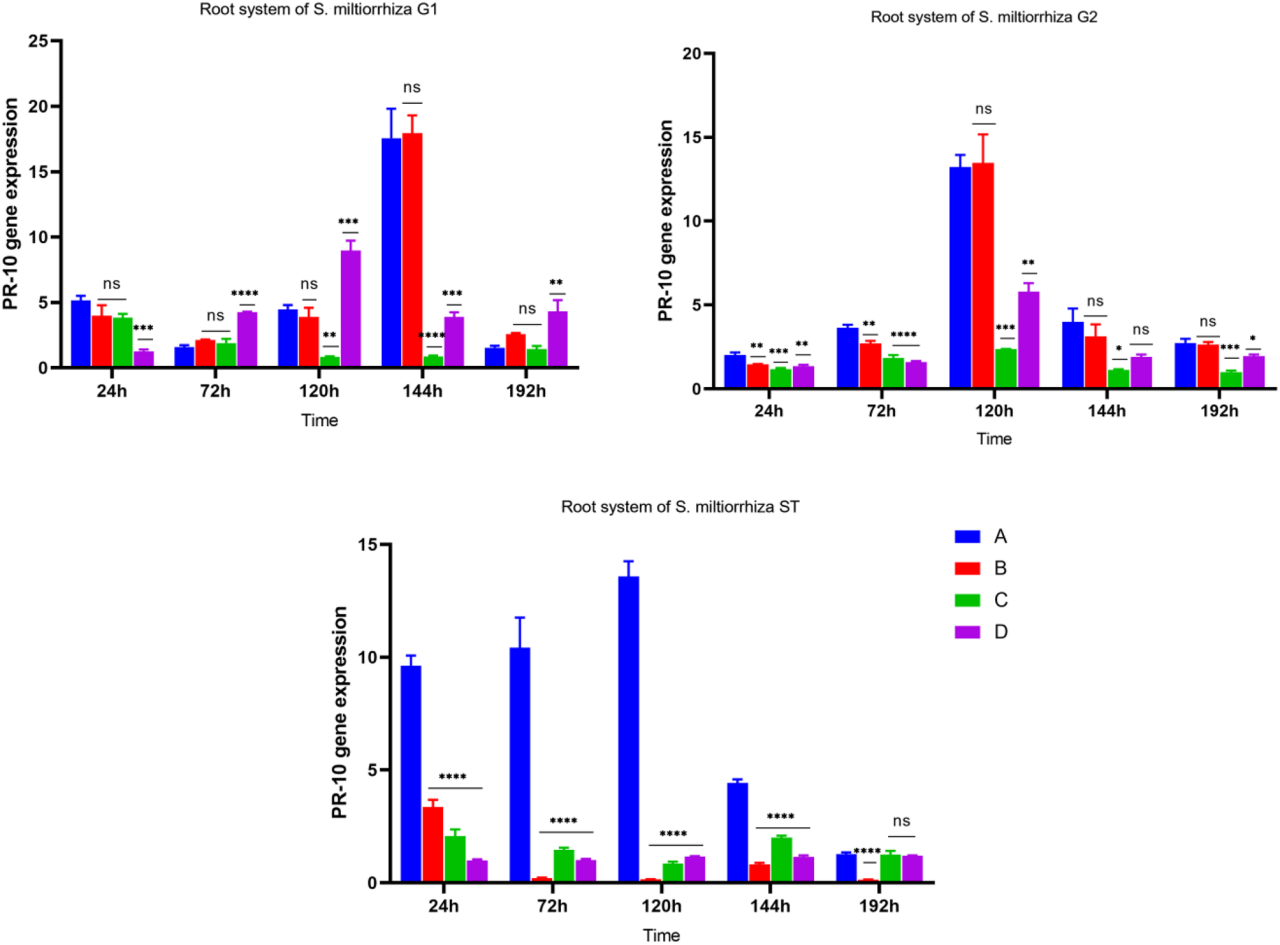
*PR-10* gene expression of four groups. Note: ****p ≤ 0.0001, ***p ≤ 0.001, **p ≤ 0.01, *p ≤ 0.05, ^ns^*p* > 0.05.

**Figure 12:**
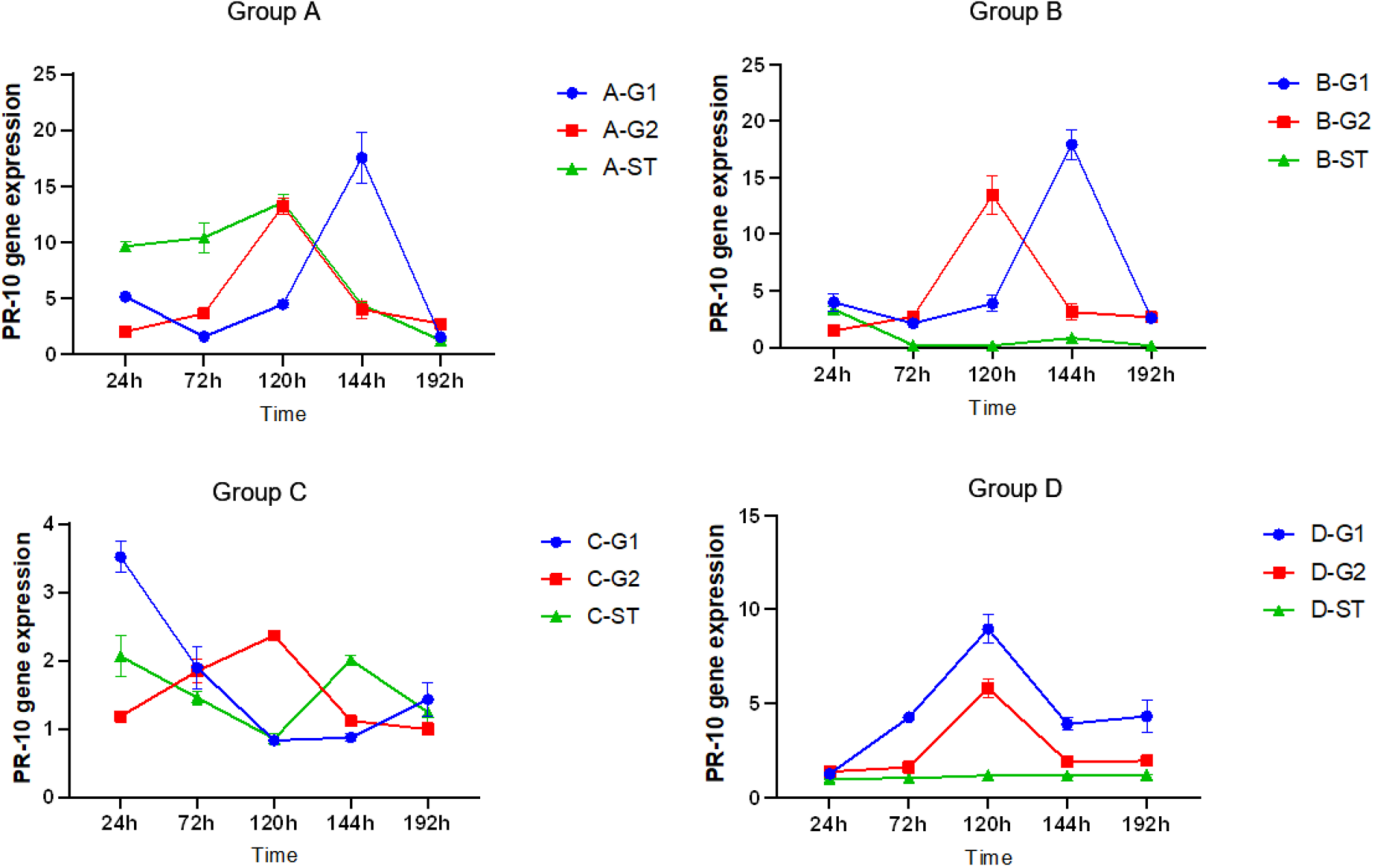
Changes in root *PR-10* gene expression in each group.

According to the detection of fluorescence quantitative PCR technology, as shown in Figs. 11 and 12, at 144 h, the *PR-10* gene had the highest expression level in the A-G1 root system of *S. miltiorrhiza*, much higher than that in the root system of *S. miltiorrhiza* treated only with pathogenic fungi without AM fungi. Only *S. miltiorrhiza* inoculated with *Fusarium solani* could induce the expression of the *PR-10* gene at 72 h, and the gene expression reached the highest at 120 h. For the roots of *S. miltiorrhiza* inoculated with *Fusarium solani*, the amount of gene changes showed a trend of first increasing and then decreasing. The expression of the *PR-10* gene changed in different groups. The highest expression of A-G1 was 2.16 times that of D-G1; The expression level of *PR-10* gene in the root system of C-G1 *S. miltiorrhiza*, which was only treated with AMF fungi, was similar to that of D-ST, which was not treated with both, and the expression level of *PR-10* gene in C-G1 increased only after 24 h. Due to the absence of pathogenic fungal infection in C-G1, the defense mechanism for disease resistance was not induced, and the gene expression level of *PR-10* showed a low level of change.

In the healthy root system on the other side of the *S. miltiorrhiza* donor, for *S. miltiorrhiza* inoculated with AM fungi and *Fusarium solani*, the *PR-10* gene showed a trend of first increasing and then decreasing. The *PR-10* gene was gradually induced to express in 24 h-120 h, and the gene expression reached the highest point in 120 h. For *S. miltiorrhiza* inoculated with *Fusarium solani* only, the expression of the *PR-10* gene also showed a trend of increasing first and then decreasing and reached the highest expression at 120 h, but the expression of A-G2 *S. miltiorrhiza* root system inoculated with double inoculation was 2.28 times of its expression. The G1 roots of *S. miltiorrhiza* inoculated only with AM fungi were not infected by *Fusarium solani*, and there was no transmission of disease resistance signal. The expression of *PR-10* gene changed steadily and was at a low level.

In the root system of recipient *S. miltiorrhiza*, there were roots connected by hyphal network with donor *S. miltiorrhiza*. After the pathogen *Fusarium solani* was inoculated into donor *S. miltiorrhiza* G1, the expression of *PR-10* gene showed a trend of first increasing and then decreasing, and reached the highest level at 120 h. However, the root G1 was only inoculated with *S. miltiorrhiza* of *Fusarium solani*, because no AM fungi were applied, and there was no hyphal network connection between donor *S. miltiorrhiza* and recipient *S. miltiorrhiza*, the expression of *PR-10* gene in D-ST root system remained unchanged, and the highest expression of A-ST was 11.25 times of its highest expression.

The above data show that the expression of *PR-10* gene can only be induced after inoculation of *S. miltiorrhiza* with the pathogenic fungi *Fusarium solani*. In *S. miltiorrhiza* without inoculation of the pathogenic fungi *Fusarium solani*, the *PR-10* gene expression of *S. miltiorrhiza* inoculated with AM fungi and without AM fungi has always been low.Under the condition that the recipient *S. miltiorrhiza* in group A was not directly infected by the pathogen fungi, the *PR-10* gene showed a high expression, which was different from that of the other three groups. This showed that the hyphal network between the recipient and the donor could transmit the signal of resistance to root rot. after the infection of *Fusarium solani*, the defense response of *S. miltiorrhiza* was rapidly activated, and the relevant disease resistance signal was transmitted to the recipient *S. miltiorrhiza* through the hyphal network, making it start the relevant defense response in advance, In response to the upcoming coercion.

The symbiosis between arbuscular mycorrhizal fungi and plants forms a mycorrhizal hyphal network, which is a reciprocal effect for plants and fungi. The formation of mycorrhizas not only increases the absorption of mineral elements in the soil, but also improves the disease resistance of plants^33^. This study proved that the defense response of *S. miltiorrhiza* inoculated with AM fungi was rapidly activated when the plant was subjected to disease stress. Compared with *S. miltiorrhiza* not inoculated with AM fungi, the increased expression level of defense gene *PR-10* can enhance the disease resistance of *S. miltiorrhiza*.

#### Induced expression of ABA signal transduction-related gene *SnRK2* in the roots of donor and recipient *S. miltiorrhiza*

**Figure 13:**
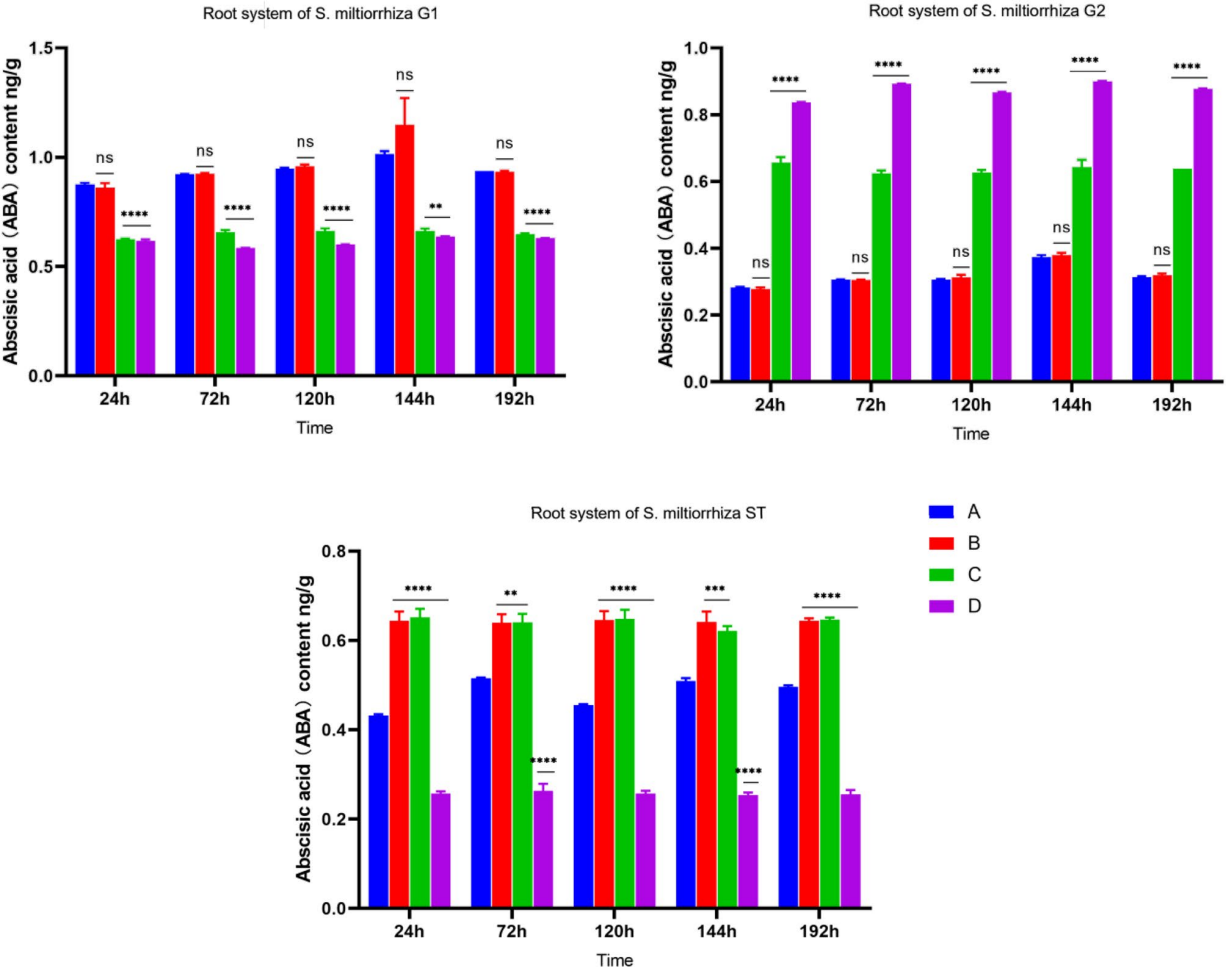
ABA content of four groups. ****p ≤ 0.0001, ***p ≤ 0.001, **p ≤ 0.01, ^ns^*p* > 0.05.

There was also a significant difference in ABA content between B-ST inoculated with AM fungi and D-ST not inoculated with AM fungi. According to the comparison of ABA content in Figs. 4 and 13, we can get that the ABA content of *S. miltiorrhiza* inoculated with AM fungi is 2.50 times that of uninoculated. Meanwhile, the content of ABA varied between recipients and donors in the four treatment groups, which was not the same as JA and SA.

Therefore, when no pathogenic fungi were applied to *S. miltiorrhiza*, the ABA content of B-ST inoculated with AMF fungi is higher than that of D-ST not inoculated with AMF fungi, indicating that AM fungi can promote the accumulation of ABA content in *S. miltiorrhiza*. The ABA content of C-G1, which was only applied with AM fungi and not inoculated with pathogenic fungi, had not changed significantly, but after the application of pathogenic fungi to *S. miltiorrhiza*, the ABA content of *S. miltiorrhiza* had interesting changes.

**Figure 14:**
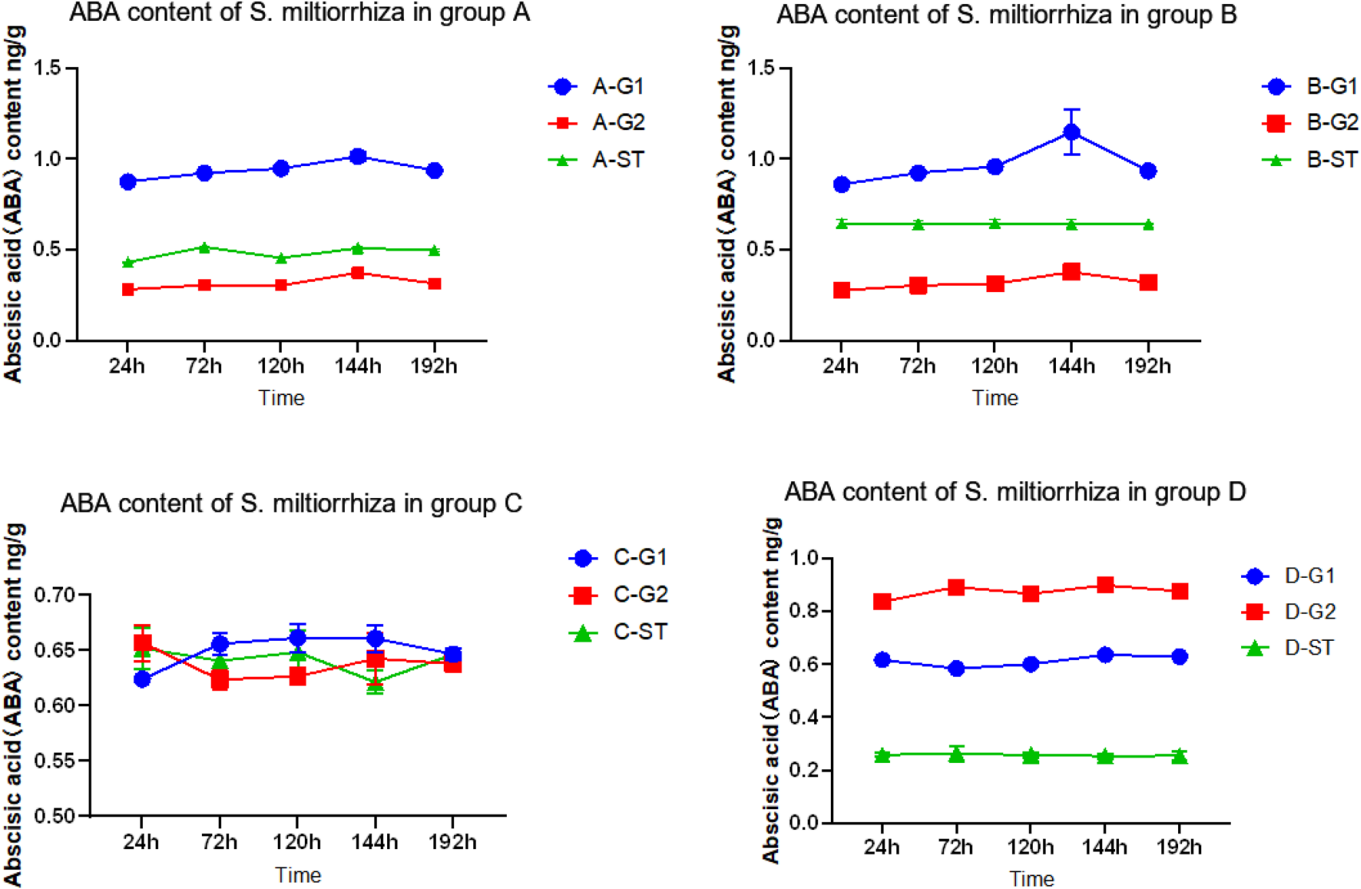
Changes in root ABA content in each group.

As shown in Fig. 14, in treatment group A, the ABA content of A-G1 increased first and then decreased, but the change was not obvious, and its content was greater than that of the other groups. Although D-G1 was not inoculated with AM fungi, it may be that *Fusarium solani* was applied at the G1 end, so the content was also high. The opposite phenomenon occurred in the G2 root system of *S. miltiorrhiza*^34^. The ABA content of the D-G2 root system was always at the highest level, which may be because *S. miltiorrhiza* without AM fungi was not resistant to disease stress, but when it was stressed by pathogenic fungi, *S. miltiorrhiza* might enhance its disease resistance by controlling its own stomatal movement and water content changes. The study showed that the closure of stomata and the reduction of ion permeability are conducive to plants' resistance to the invasion of fungi, and when plants are stressed by bacteria, fungi, and viruses, the endogenous ABA level of plants will increase^35^. The low ABA content in A-G2 may be due to the application of AM fungi by *S. miltiorrhiza*, which improved the resistance of *S. miltiorrhiza* through symbiosis when no stress occurred, so the ABA content in the root system of *S. miltiorrhiza* G2 did not significantly increase during this period. The data of recipient *S. miltiorrhiza* showed that the ABA content of A-ST roots was lower than that of B-ST. Increasing the content of ABA and jasmonic acid can improve plant biological activity^36^ and enhance plant resistance to insect pests^37^, but many evidences also indicate that ABA and jasmonic acid signaling pathway may antagonize each other. In the study of resistance to cabbage clubroot disease, it was found^38^. When exogenous hormone MeJA was applied to cabbage, the JA content of the cabbage itself increased, while the ABA content decreased. So the ABA content of donors in group A was lower than that of donors in group B, probably because the JA content of donors in group A was significantly increased, which inhibited the synthesis and accumulation of ABA.

As the most important stress resistance hormone of plants, ABA has the functions of promoting plant dormancy, reducing plant water loss, inhibiting plant growth and development^39^, inducing the expression of stress-related genes^40^, etc., which can promote the adaptation of plants to the stress environment. Nowadays, studies have shown that the ABA signaling system also interacts with many hormone pathways during transcription^41–43^. Our research group completed the sequencing of the transcriptome of *S. miltiorrhiza* symbiosis with mycorrhiza in the previous experiment and screened the resistance gene *SnRK2*. As a gene related to the self-resistance response of *S. miltiorrhiza*, *SnRK2* type protein kinase can phosphorylate arebs / ABFs and transcription factor ABI5, and then induce the expression of the target gene through arebs in the promoter, promoting ABA response^44^.

**Figure 15:**
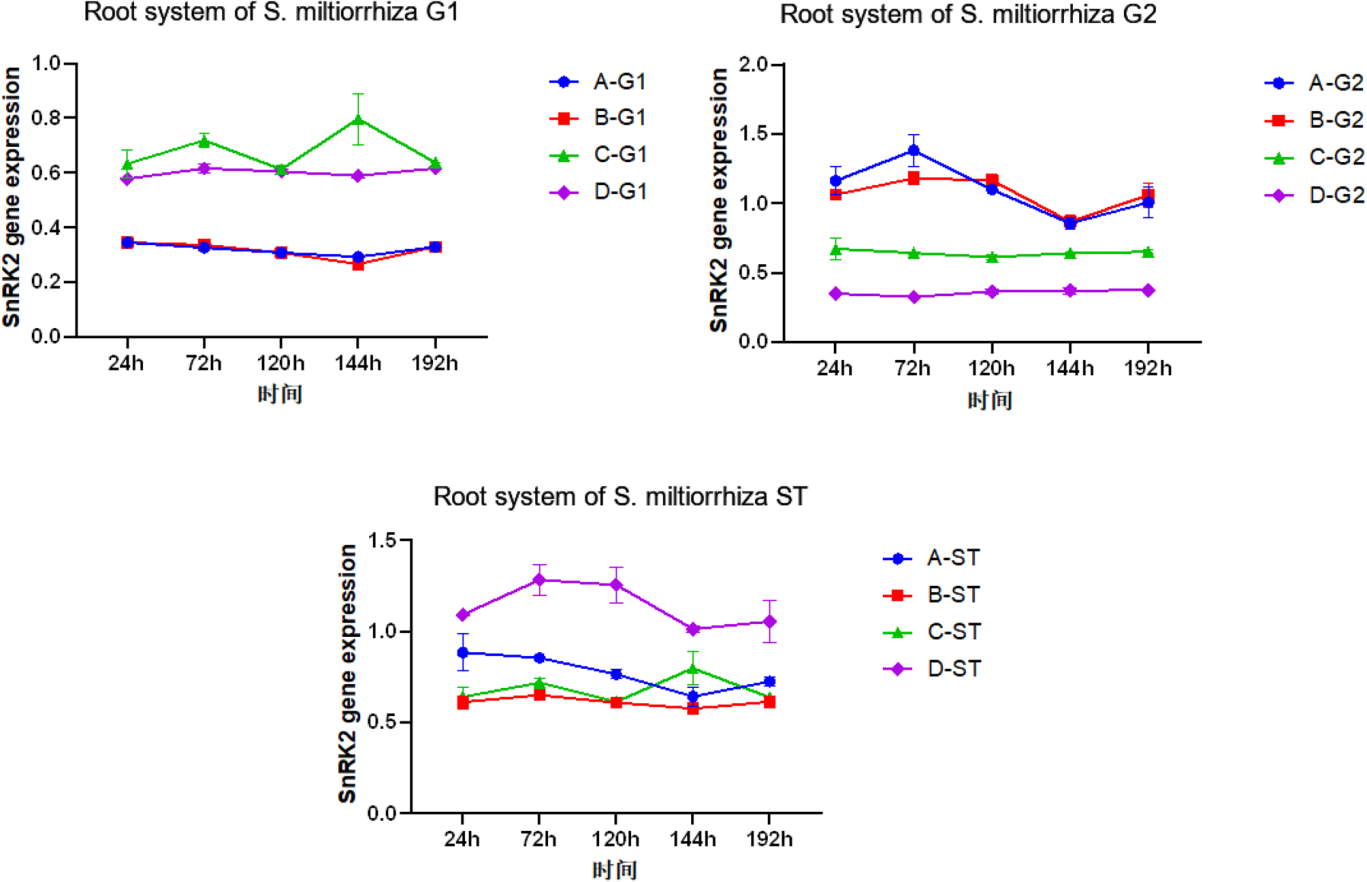
Changes in *SnRK2* gene expression between miltiorrhiza donors and recipients.

**Figure 16:**
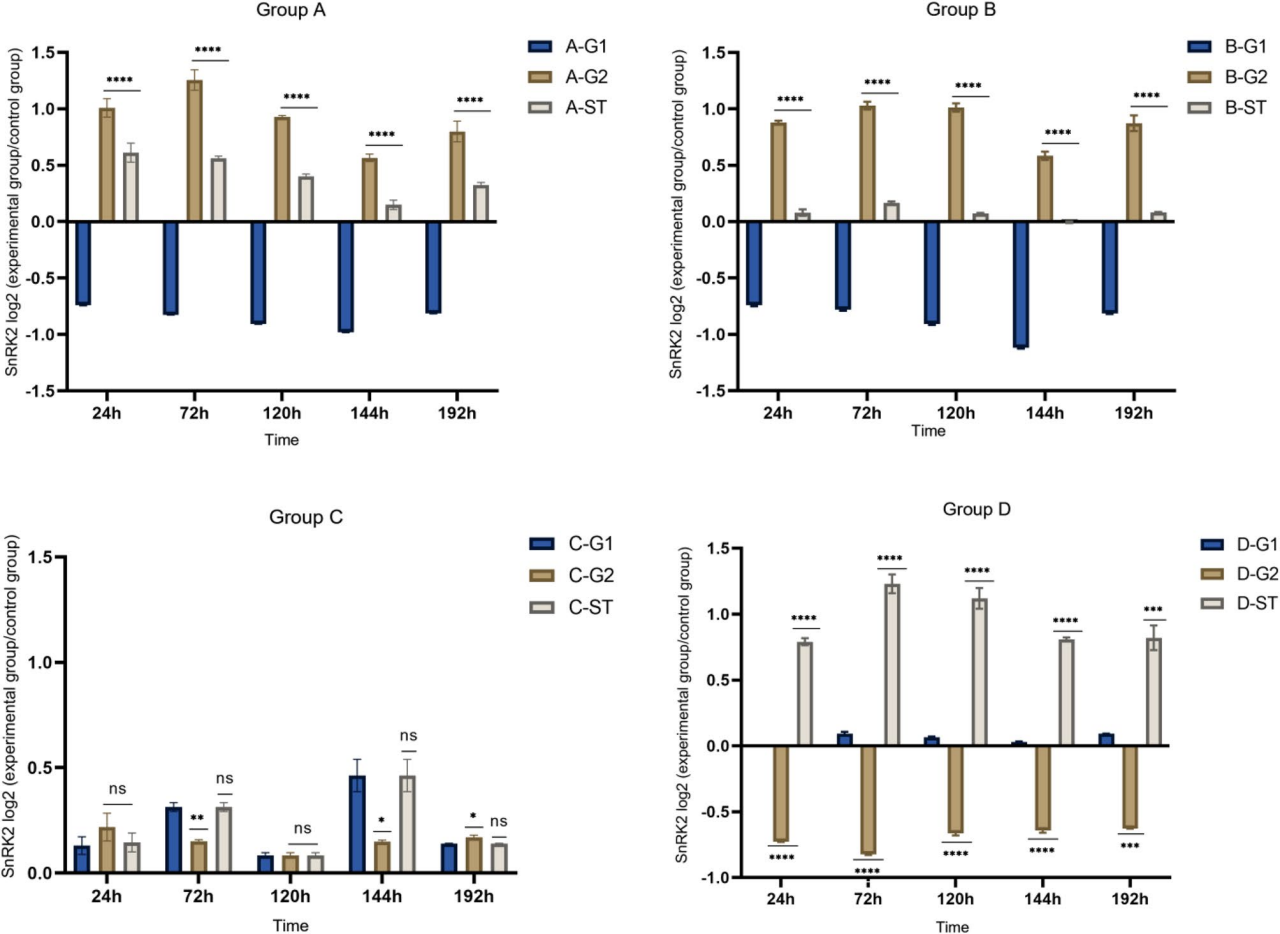
*SnRK2* gene changes in each group. ****p ≤ 0.0001, ***p ≤ 0.001, **p ≤ 0.01, *p ≤ 0.05, ^ns^*p* > 0.05.

As shown in Figs. 15, 16, *SnRK2* gene changes were also different in the four *S. miltiorrhiza* treatment groups with different treatment methods. It can be seen that for the treatment group D without AM fungi, the expression of the *SnRK2* gene is up-regulated relative to the other three groups with AM fungi, which is consistent with our results obtained in transcriptome sequencing. If there is no communication between the *S. miltiorrhiza* donor and the recipient, the gene expression of group A recipients should be consistent with that of group B recipients. If the change of gene expression is caused by mycorrhizal infection, the receptor *S. miltiorrhiza* in group A and group C should have the same change, but relative to the receptors in group B and group D, the expression of A-ST is higher than that of b-st and lower than that of D-ST, which indicates that there is one or more transmission of disease resistance signals between the donor *S. miltiorrhiza* and the recipient *S. miltiorrhiza* connected by the hyphal network, which makes the *SnRK2* gene of *S. miltiorrhiza* make corresponding changes.

The data results of the detection of plant endogenous hormones JA, SA, and ABA showed that after inoculation with pathogenic fungi, JA showed antagonistic changes with SA and ABA, the increase of JA content inhibited the expression of SA and ABA hormones, *SnRK2* was inhibited by ABA in the signal transduction pathway, so we speculated that JA might positively regulate the expression of *SnRK2* gene. When the root of *S. miltiorrhiza* is subjected to stress, the JA content increases, and the *SnRK2* gene is positively regulated by it, thereby inhibiting ABA signal transduction in the plant body, reducing the inhibition of root growth, promoting the decomposition of proteins, sugars, and other substances in the plant body, and improving the plant's resistance.

## Conclusion and discussion

In nature, abiotic stress and biotic stress are the main threats to plant growth and development. At present, most studies are aimed at abiotic stresses, such as drought stress^45^. Plants need water for growth. For their growth and development, plants have formed a mechanism to resist drought stress after long-term evolution^46^. We have little knowledge about the signaling mechanisms by which plants respond to abiotic stress, but in this experiment, we found that plants respond much faster to pathogenic fungi when containing mycorrhizal hyphal networks than we imagine.

This research shows that plants and plant roots can form a huge mycorrhizal hyphal network through the hyphal bridge between the soil. When the plants on one side are given drought stress, the plants can sense the stress signal in advance, and then prepare for drought and drought resistance in advance, which is conducive to the plants' resistance to the adverse environment. Biological stress is mainly caused by various diseases, such as leaf spots^47,48^, blight^49^, etc. Fruitful research achievements have been made in model plants and important crops such as Arabidopsis, tobacco, rice, soybean, corn, etc., but the research on disease resistance signal transmission of medicinal plants is still relatively backward. This experiment to some extent compensates for this deficiency.

After plants and AM fungi form symbionts, the cell structure in plant roots changes and is rich in abundant Arbuscular structures^50^. It is the existence of Arbuscular structures that makes the communication between plants and fungi more favorable. Through hyphal connection, it can realize distant communication between plants. The mycorrhizal structure enables plants to transmit signals to adjacent roots faster when they encounter stress^51^.

This experiment confirmed that the inoculation of AM fungi can increase the content of JA, SA, and ABA in *S. miltiorrhiza*. JA and SA are important signal molecules in plants. The increase in hormone content can enhance the ability of plants to resist external stress. In the analysis of *PR-10* gene expression, it was found that *S. miltiorrhiza*, which formed a mycelial network between AM fungi and plant roots, could promote healthy roots to transmit disease resistance signals to recipient *S. miltiorrhiza* through mycelial bridges when infected by pathogenic fungi. The *SnRK2* gene is down-regulated in mycorrhizal symbiotic plants. When the pathogenic fungi threaten the donor plants, it will also cause a series of corresponding changes in the receptor plant *SnRK2* gene: ABA accumulation inhibits the expression of *SnRK2*, and *SnRK2* is in the ABA transduction pathway. When *SnRK2* is inhibited, it weakens the ABA signal transduction in the root, resulting in ABA reducing the inhibition of root growth and other biological processes, which may be the response feedback of ABA hormone signal to AM fungi and pathogenic fungi.

*S. miltiorrhiza* can transmit the signal of resistance to root rot through the jasmonic acid pathway; When plants suffer from disease stress, the content of JA increases significantly, and the increase of JA content will inhibit the content of SA in plants; The gene expression of *PR-10* gene in the roots of *S. miltiorrhiza* with arbuscular mycorrhizal network infected by pathogenic fungi was 17.56 times higher than that inoculated only with pathogenic fungi; Changes in hormone content will also cause changes in the expression of related defense genes, such as *SnRK2* is inhibited by ABA in the signal transduction pathway, while JA and ABA show antagonistic changes after inoculation of pathogenic fungi in *S. miltiorrhiza*, so JA may positively regulate the expression of *SnRK2* gene.

With the development of ecological agriculture, more and more local planting enterprises begin to pay attention to environmental protection and the good quality of products. In future agriculture, the use of AM fungi for biological prevention and control has a good industry prospect. Therefore, modern scientific and technological means should be used to promote the greening of agriculture and develop green agriculture in the future.

### Supplementary Information

Supplementary Information.

## Data Availability

The datasets used and analysed during the current study available from the corresponding author on reasonable request.
