# Supplementary material for: Study on signal transmission mechanism of arbuscular mycorrhizal hyphal network against root rot of Salvia miltiorrhiza
Source: Sci Rep. 2023 Oct 7;13:16936. doi: 10.1038/s41598-023-43278-5 (PMC10560300; doi:10.1038/s41598-023-43278-5)

**Supplementary material 1：**

Quality Inspection Results of Total RNA of Danshen Mycorrhiza:

As shown in the following figure, the electrophoresis bands at 28S and 18S are clear and bright, indicating that the extracted total RNA sample has good quality and high integrity. The concentration and A260/A280 ratio of total RNA were measured using the Nano-300 micro spectrophotometer instrument. The A260/A280 ratio of the sample is between 1.9 and 2.0, indicating that the purity of the total RNA extracted in this experiment mostly meets the requirements. Use high-quality RNA for subsequent experiments.


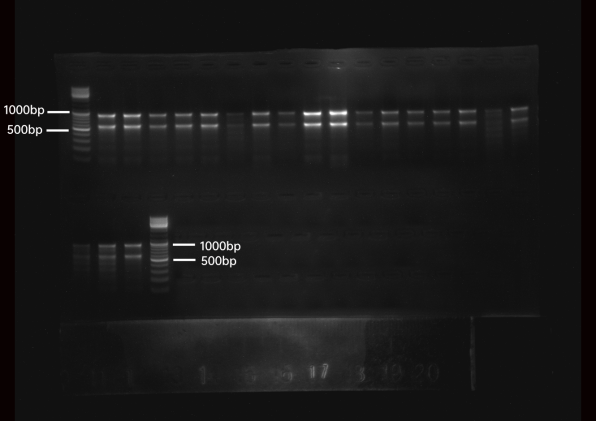

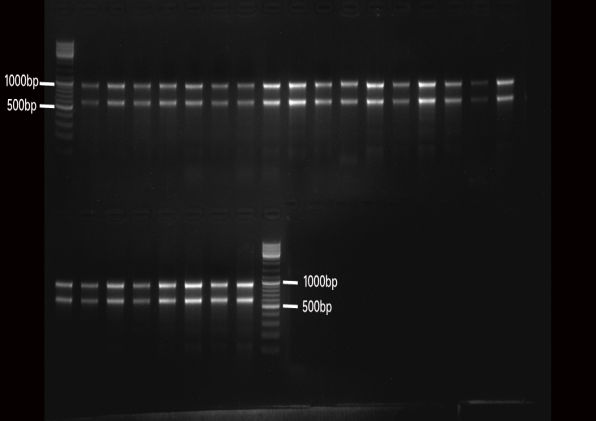


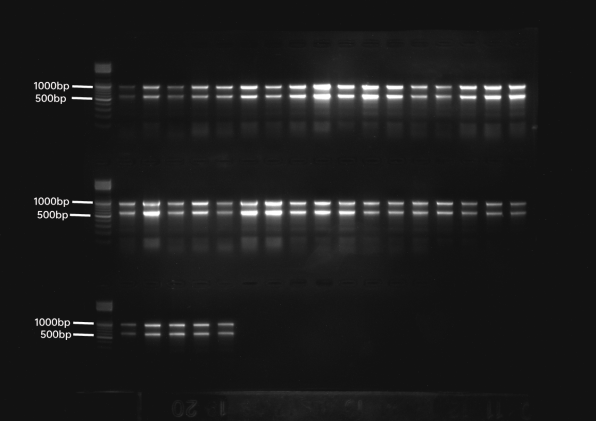

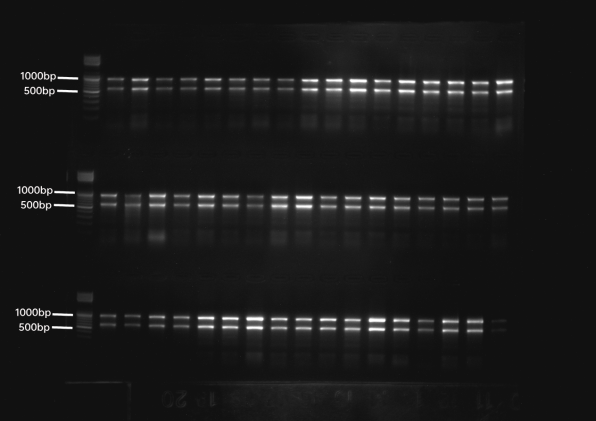


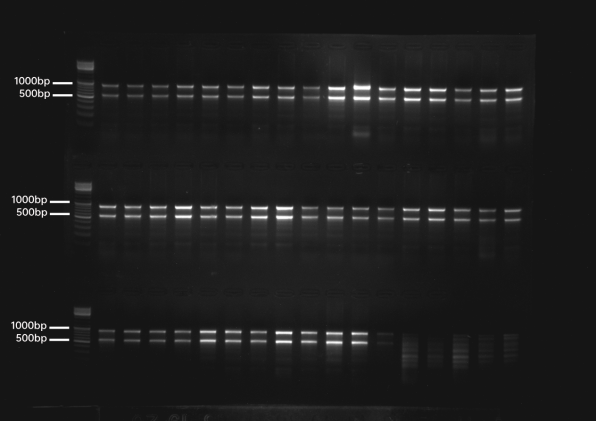

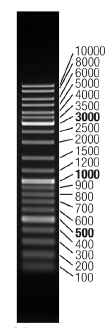


Agarose gel electrophoresis diagram

Notes: Marker is a DNA marker that only detects genomic contamination and does not represent RNA band size.

**Supplementary material 2:**

Melting curve of *PR-10* gene:


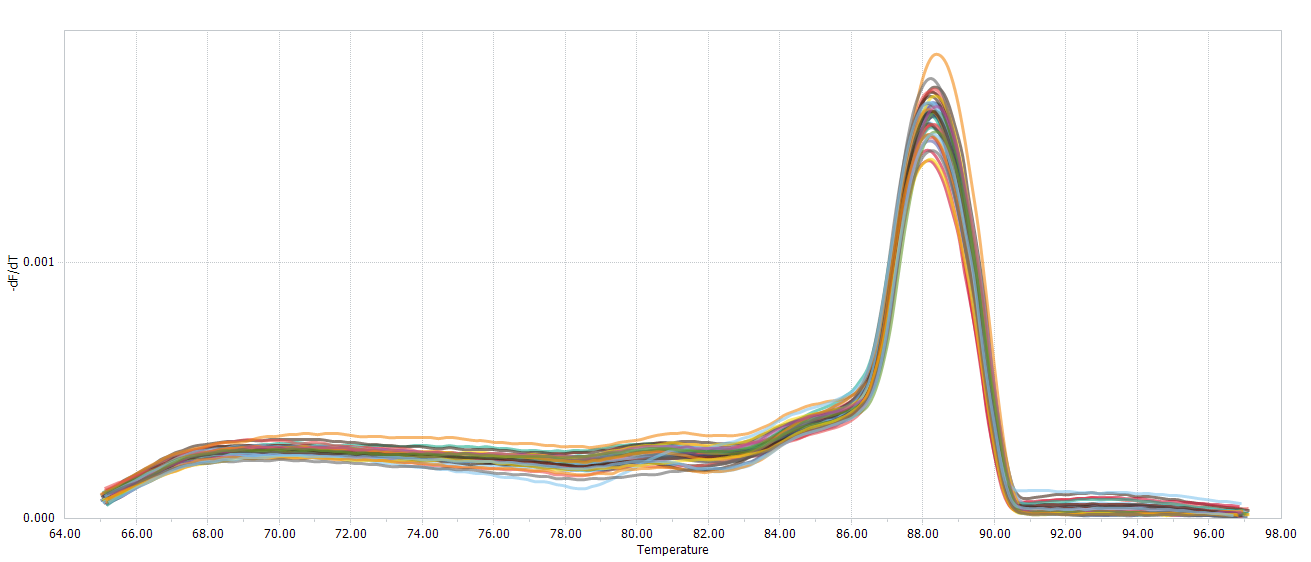


Melting curve of *SnRK2* gene:


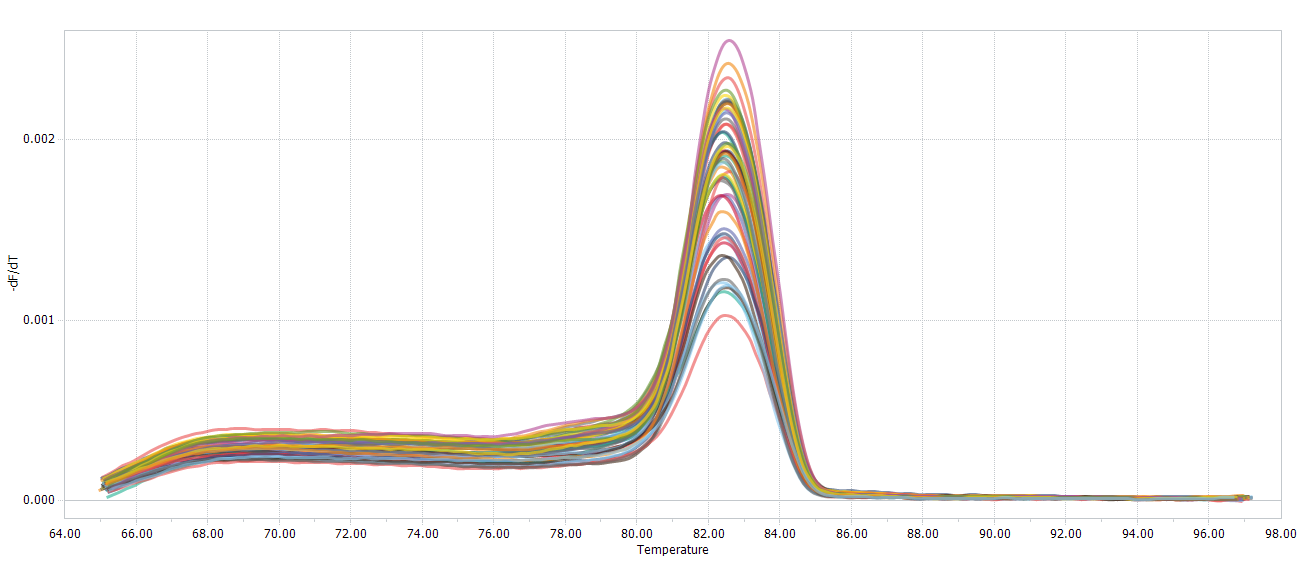


Melting curve of *Actin* gene:


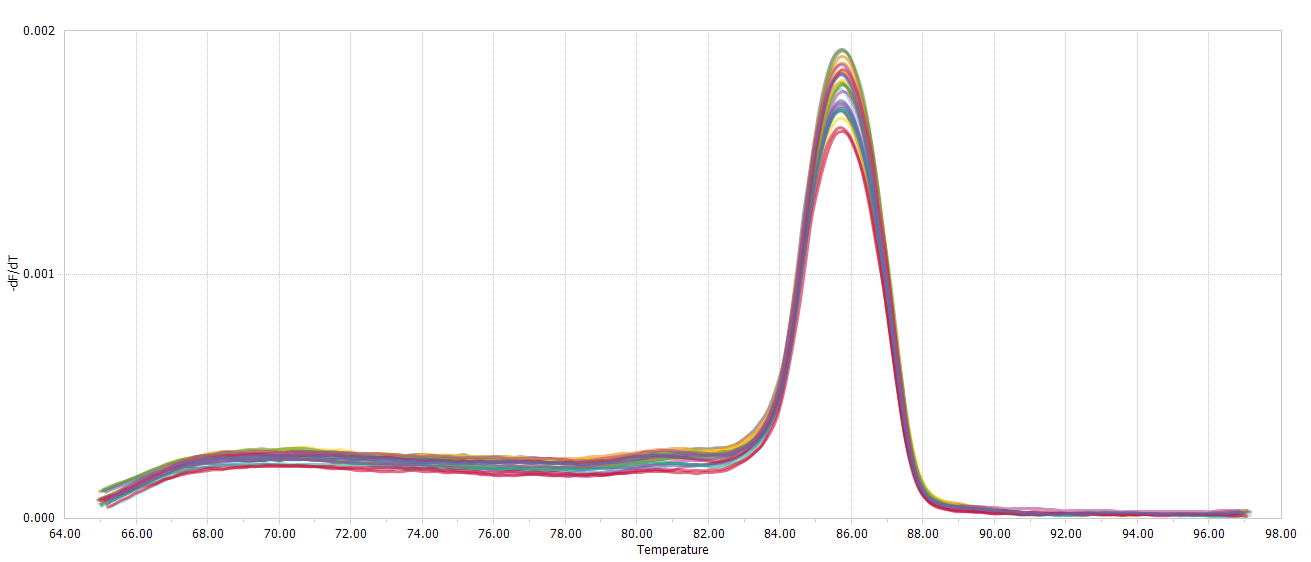

Supplement: Supplementary file 1 — Supplementary Information. [file 41598_2023_43278_MOESM1_ESM.docx]
